# Two proteolytic fragments of menin coordinate the nuclear transcription and postsynaptic clustering of neurotransmitter receptors during synaptogenesis between *Lymnaea* neurons

**DOI:** 10.1038/srep31779

**Published:** 2016-08-19

**Authors:** Angela M. Getz, Frank Visser, Erin M. Bell, Fenglian Xu, Nichole M. Flynn, Wali Zaidi, Naweed I. Syed

**Affiliations:** 1 Department of Cell Biology & Anatomy, Hotchkiss Brain Institute and Alberta Children’s Hospital Research Institute, University of Calgary, Calgary, Alberta, T2N 1N4, Canada; 2 Department of Neuroscience, Hotchkiss Brain Institute, University of Calgary, Calgary, Alberta, T2N 1N4, Canada; 3 Department of Physiology & Pharmacology, Hotchkiss Brain Institute, University of Calgary, Calgary, Alberta, T2N 1N4, Canada; 4 Department of Biology, Saint Louis University, Saint Louis, Missouri, 63103, USA

## Abstract

Synapse formation and plasticity depend on nuclear transcription and site-specific protein targeting, but the molecular mechanisms that coordinate these steps have not been well defined. The *MEN1* tumor suppressor gene, which encodes the protein menin, is known to induce synapse formation and plasticity in the CNS. This synaptogenic function has been conserved across evolution, however the underlying molecular mechanisms remain unidentified. Here, using central neurons from the invertebrate *Lymnaea stagnalis*, we demonstrate that menin coordinates subunit-specific transcriptional regulation and synaptic clustering of nicotinic acetylcholine receptors (nAChR) during neurotrophic factor (NTF)-dependent excitatory synaptogenesis, via two proteolytic fragments generated by calpain cleavage. Whereas menin is largely regarded as a nuclear protein, our data demonstrate a novel cytoplasmic function at central synapses. Furthermore, this study identifies a novel synaptogenic mechanism in which a single gene product coordinates the nuclear transcription and postsynaptic targeting of neurotransmitter receptors through distinct molecular functions of differentially localized proteolytic fragments.

The *MEN1* (Multiple Endocrine Neoplasia Type 1) gene, which encodes the protein menin, is a tumor suppressor that has been well conserved across evolution. However, the absence of functional domains, structural motifs, or significant regions of homology to any known genes or proteins affords little insight into the evolutionary origins of *MEN1* and the molecular function of menin^1^. Menin is thought to be primarily a nuclear protein, targeted by nuclear localization signals (NLS) in the carboxyl (C)-terminus^2^. In the nucleus, menin acts as a molecular scaffold to bind a range of transcriptional activators and repressors to control gene expression in response to numerous cell signaling pathways^3^,^4^. In addition to roles as transcriptional regulators, many tumor suppressors are known to act as cytoplasmic molecular scaffolds to integrate cell-cell signaling events^5^, including synaptogenesis^6^. Nuclear exit signals (NES) in the amino (N)-terminus have also been found to shuttle menin into the cytoplasm^7^, although its role here remains poorly understood.

Our group was the first to identify a synaptogenic role for *MEN1* with a well-conserved orthologue from the invertebrate mollusk *Lymnaea stagnalis*. We have previously reported that neurotrophic factor (NTF)-mediated expression of *Lymnaea-MEN1* (*L-MEN1*/*L-*menin, described in subsequent text as *MEN1*/menin) is necessary and sufficient for *in vitro* and *in situ* synapse formation between *Lymnaea* neurons^8^,^9^. Other studies from murine models have demonstrated that menin induces plasticity in the spinal cord dorsal horn to produce neuropathic pain in response to peripheral nerve injury^10^,^11^,^12^, indicating that the role for *MEN1* in CNS synapse formation and plasticity has been conserved across evolution. Despite prevalent *MEN1* expression in both the developing and adult central nervous system (CNS)^13^, the molecular function of menin in neurons remains, however, unidentified.

In the present study, we took advantage of a *MEN1*-dependent excitatory cholinergic synapse between *Lymnaea* CNS neurons to pursue identification of the molecular mechanisms underlying the synaptogenic effect of menin. Here we report that menin is cleaved at an evolutionarily conserved calpain site in response to NTF signaling. The resulting menin proteolytic fragments coordinate nuclear and synaptic events necessary for excitatory synaptogenesis, including subunit-selective nicotinic acetylcholine receptor (nAChR) gene induction via the N-terminal fragment, and postsynaptic clustering of nAChR via the C-terminal fragment. This study (i) is the first to demonstrate that menin is cleaved by calpain, (ii) characterizes a role for menin in the transcriptional regulation of neuronal nAChR, (iii) identifies a novel cytoplasmic function for menin in mediating cell-cell interactions, and (iv) identifies a novel synaptogenic mechanism in which a single gene product coordinates the nuclear transcription and postsynaptic targeting of neurotransmitter receptors.

## Results

### Menin is localized to a synapse between *Lymnaea* neurons

The *in vitro* reconstructed synapse between the *Lymnaea* presynaptic visceral dorsal 4 interneuron (VD4) and its postsynaptic target left pedal dorsal 1 (LPeD1) is a well-characterized model for the study of both NTF- and *MEN1*-dependent synaptogenesis. VD4-LPeD1 forms an inhibitory cholinergic synapse in the absence of NTF (cultured in defined media; DM), and an excitatory cholinergic synapse in the presence of NTF (cultured in CNS-conditioned DM; CM, NTF-rich). While menin is expressed in both pre- and postsynaptic neurons, only postsynaptic expression is required for synaptogenesis. Excitatory cholinergic synapse formation between VD4-LPeD1 in CM is inhibited by *MEN1* knockdown in LPeD1^8^, and can also be induced in DM by *MEN1* expression in LPeD1^9^. Our previous observations therefore suggest that the synaptogenic function of *MEN1* may be specific to the regulation of neurotransmitter receptors, although how this occurs remains unknown.

**Figure 1:**
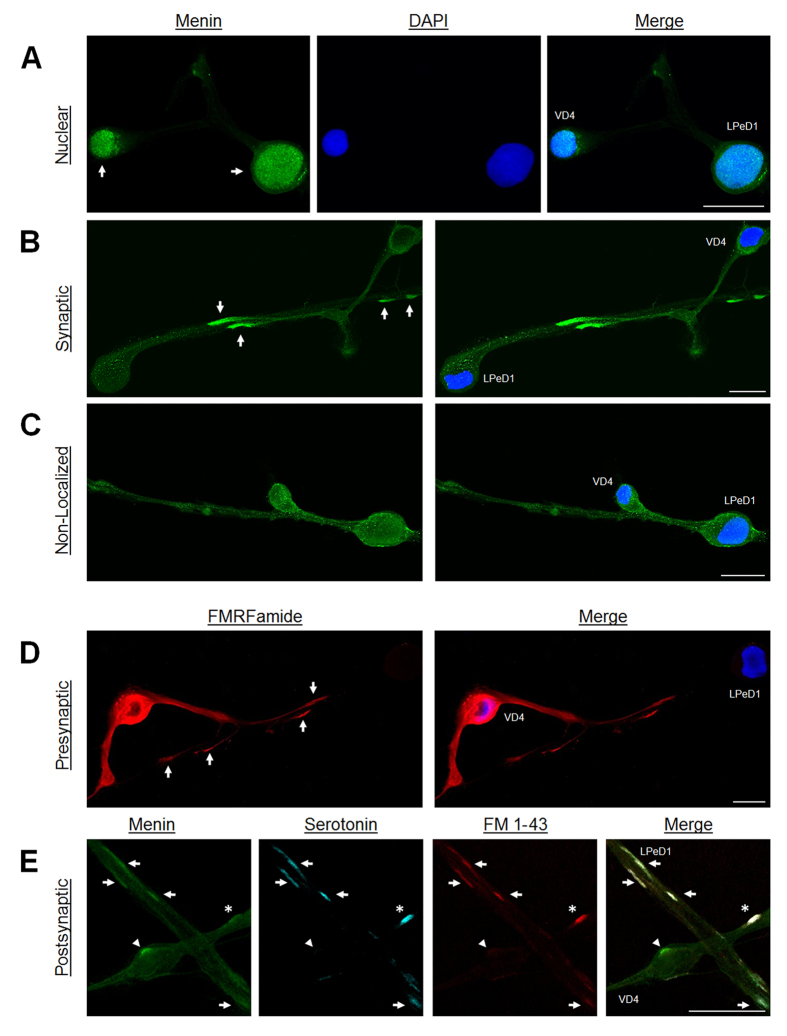
Postsynaptic clustering of menin in *Lymnaea* neurons. ICC characterization of VD4-LPeD1 axon-axon pairs cultured in CM. (**A**) Nuclear localization of menin (n = 5/28). The nucleus was confirmed with DAPI. Arrows, nuclei. (**B**) Synaptic localization of menin (n = 19/28). Arrows, synaptic sites. (**C**) Non-localized menin (n = 4/28). (**D**) ICC localization of FMRFamide (VD4, presynaptic), shown to illustrate presynaptic innervation (n = 4). Arrows, presynaptic sites. (**E**) Co-localization of FM 1-43 (VD4, presynaptic) and serotonin (5-HT; LPeD1, postsynaptic) to verify that menin clustering occurs at synaptic sites (n = 4). Menin puncta at synaptic sites (FM 1-43 and 5-HT positive) are observed along both the LPeD1 axon (arrows) and the VD4 axon (asterisk), due to reciprocal outgrowth of synaptic processes. Menin puncta at non-synaptic sites (FM 1-43 and 5-HT negative) are observed in VD4 (arrowhead), but not LPeD1, suggesting that the source of the synaptic menin signal is postsynaptic. Scale bars, 50 μm.

12 nAChR subunits have been identified in *Lymnaea* (*L-*nAChR A-L, described in subsequent text as nAChR A-L), which form excitatory or inhibitory nAChR (cation- or anion-selective channels)^14^. Given that menin is described as a nuclear protein in the cancer literature^2^, our initial hypothesis was that menin induces the transcriptional upregulation of excitatory nAChR subunits to mediate excitatory synaptogenesis in response to NTF. Therefore, we first sought to characterize the subcellular localization of menin in neurons. VD4-LPeD1 were cultured in an axon-axon configuration in CM, and immunocytochemistry (ICC) was performed using a commercial antibody against a well conserved menin C-terminal epitope. Contrary to our expectations, few neurons showed primarily nuclear localization of menin (Fig. 1A; n = 5/28, 18%). The majority of pairs exhibited a presumptive synaptic localization (Fig. 1B; n = 19/28, 68%), whereas the remainder showed primarily cytoplasmic, non-localized distribution (Fig. 1C; n = 4/28, 14%). We next sought verify that the axonal clustering of menin depicts synaptic recruitment. To this end, we first labeled FMRFamide neuropeptides expressed in VD4, but not LPeD1, to visualize presynaptic innervation of the LPeD1 axon. Menin immunoreactivity (see Fig. 1B) mimics the axonal distribution patterns of FMRFamide immunoreactivity (Fig. 1D; n = 4), suggesting that menin clustering occurs at innervated sites along the LPeD1 axon. Secondly, to confirm that menin clustering occurs at functional synaptic sites, presynaptic active zones in VD4 were loaded with the membrane impermeant dye FM 1-43 by depolarization-induced synaptic vesicle recycling, and ICC was then used to label serotonin (5-HT; the neurotransmitter used by LPeD1) at the postsynaptic membrane, as well as menin (Fig. 1E; n = 4). Multiple sites of co-localization were observed, indicating that menin clusters to functional synaptic sites where the presynaptic active zone (FM 1-43 positive) is opposed by the postsynaptic membrane (5-HT positive). Menin positive, but FM 1-43 and 5-HT negative sites in VD4 indicate that presynaptic menin does not cluster to synaptic sites. Menin puncta in LPeD1, however, were all FM 1-43 and 5-HT positive, and it is therefore likely that the synaptic menin signal represents postsynaptic recruitment. These data suggest that there is a previously unidentified function for menin in the postsynaptic density.

### Menin is cleaved by calpain and the resulting fragments are differentially localized within neurons

**Figure 2:**
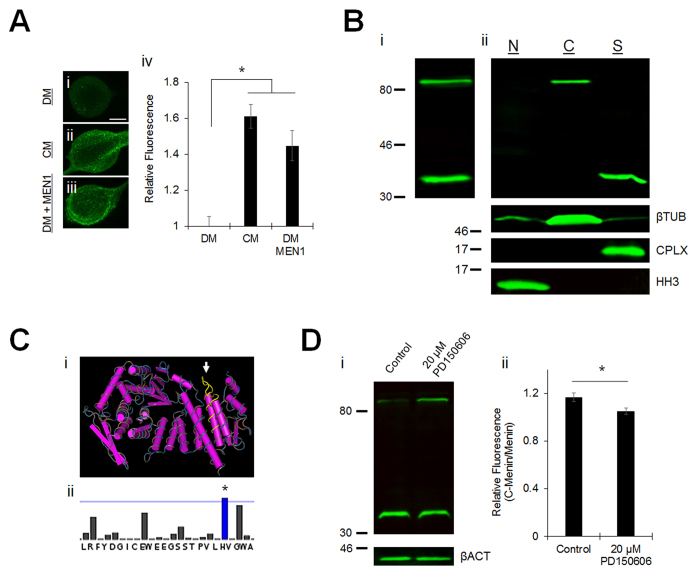
Menin is cleaved by calpain. (**A**) ICC of menin in LPeD1 cultured in DM (**i**; n = 4), CM (**ii**; n = 4), or DM + *MEN1* mRNA (**iii**; n = 4). Scale bar, 20 μm. Summary data, fold-increase in menin fluorescence, relative to DM (**iv**). Asterisk, statistical significance (one-way ANOVA), *P* < 0.05–0.001. (**B**) WB of menin (top) in a *Lymnaea* CNS whole protein sample (**i**; n = 6, representative blot) and subcellular fractions (**ii**; n = 4, representative blot). N denotes nuclear fraction, C denotes cytoplasmic fraction, S denotes synaptic fraction. β-Tubulin (βTUB), complexin (CPLX), and histone H3 (HH3) are shown to verify the subcellular fractions (bottom). (**C**) 3D structure of menin (accession no. 3U84) showing the conserved ROI (yellow, arrow), an exposed unstructured loop (**i**; see also Fig. S1). Predicted calpain cleavage site in the conserved ROI (**ii**; see also Fig. S2). Blue line indicates threshold for significant prediction scores, blue bar and asterisk indicate a significant predicted calpain cleavage site. (**D**) WB of menin in protein samples from *Lymnaea* CNS incubated in control conditions (DM + 0.1% DMSO) or in the presence of a cell-permeable calpain inhibitor (DM + 20 μM PD150606) (**i**; n = 8 each, representative blot). β-actin (βACT) is shown to verify equal loading. Summary data, calpain inhibition reduces menin cleavage (**ii**). Asterisk, statistical significance (independent t-test), *P* < 0.05.

To verify the specificity of α-menin antibody binding in *Lymnaea* preparations, we performed ICC fluorescence intensity analysis on LPeD1 neurons and Western blot (WB) analysis of protein samples from *Lymnaea* CNS. Firstly, LPeD1 neurons were cultured in DM, CM (which upregulates *MEN1* mRNA expression^9^), or DM + synthetic *MEN1* mRNA microinjection. Relative to DM, ICC fluorescence was increased in LPeD1 neurons cultured in CM or DM + *MEN1* mRNA microinjection (Fig. 2A; n = 4 each; *P* = 0.001, 0.011 respectively, one-way ANOVA; see Table S1), indicating an upregulation of menin protein expression and specificity of the α-menin signal observed in cultured *Lymnaea* neurons. Secondly, an appropriate molecular weight band was apparent for *Lymnaea* menin (84.5 kDa) in WBs, but we were also struck by the consistent presence of a more rapidly migrating band of ~40 kDa (Fig. 2Bi; n = 6, representative blot). As a broad spectrum protease inhibitor was present during sample preparations, we concluded that this lower band is likely a menin proteolytic fragment generated endogenously. We next used subcellular fractionation and WB analysis to determine the subcellular distribution of menin and the presumptive C-terminal proteolytic fragment (C-menin), reasoning that proteolytic cleavage of the epitope region might explain both the predominant absence of a nuclear α-menin signal and the predominant synaptic α-menin signal observed with ICC (see Fig. 1). CNS microsomes exhibited menin localized to the cytoplasmic fraction and the C-menin fragment to the synaptic fraction (Fig. 2Bii; n = 4, representative blot), suggesting that the synaptic localization we observed (see Fig. 1B) represents selective recruitment of the C-menin fragment.

In mammalian preparations a rapidly migrating band of ~20 kDa is routinely reported with the α-menin C-terminal epitope antibody, however this lower band is often dismissed as being due to a non-specific cross-reaction^2^. A BLAST search of the C-terminal *Lymnaea* and mammalian menin sequences detected by the C-terminal epitope antibody reveals that no other proteins bear sufficient sequence identity to indicate potential cross-reactions (data not shown). By contrast, our observations suggest that these faster migrating bands represent specific antibody binding to C-menin proteolytic fragments present in both invertebrates and vertebrates, likely generated at an evolutionarily conserved protease consensus site. Upstream of the *Lymnaea* menin sequence expansion accounting for the size difference of the lower bands we identified a region of interest (ROI) of 24 well conserved residues corresponding to an exposed unstructured loop^3^, which would be an accessible site for proteolytic cleavage (Fig. 2Ci; see also Fig. S1). We evaluated the ROI for conserved protease consensus sequences that would produce menin cleavage fragments in accordance with the observed banding patterns. We identified a putative cleavage site for the calcium-dependent protease calpain within the ROI (Fig. 2Cii; *Lymnaea* menin H^409^-V^410^ prediction score 0.31, *P* < 0.001) using a calpain substrate prediction algorithm^15^. This site is conserved in menin orthologues from *Drosophila* to human (see Fig. S2), suggesting a strong evolutionary pressure for the maintenance of menin proteolytic fragments. Calpain cleavage at this site would produce C-menin fragments with predicted molecular weights of 38 kDa (*Lymnaea*), and 19 kDa (mammalian), which are in agreement with the observed banding patterns. We next used CNS organ culture with a cell-permeable calpain inhibitor (20 μM PD150606) and WB analysis to determine whether calpain inhibition would shift band distribution towards full-length menin. Consistent with the predicted calpain cleavage site, we observed an increase in full-length menin and a corresponding decrease in the C-menin fragment upon calpain inhibition (Fig. 2D; n = 8 each; C-menin/menin relative fluorescence; DM, 1.17 ± 0.04; DM + 20 μM PD150606, 1.05 ± 0.03; *P* = 0.023, unpaired t-test).

**Figure 3:**
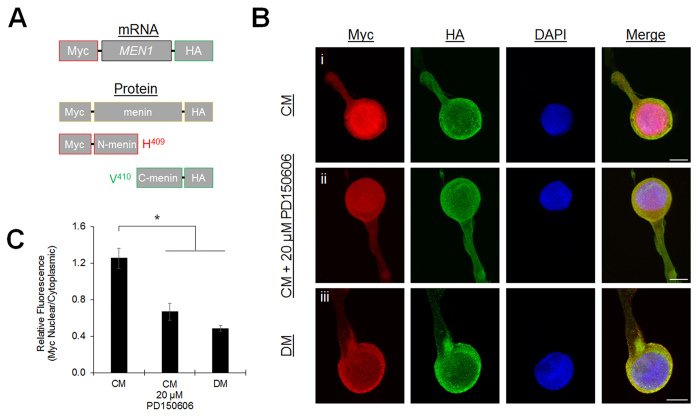
Menin fragments are differentially targeted within neurons. (**A**) Schematic of the Myc and HA epitope tagged *MEN1* mRNA construct (top) and the predicted menin protein and fragments (bottom). (**B**) ICC of Myc and HA localization in Myc-*MEN1*-HA mRNA microinjected LPeD1 neurons cultured in CM (**i**; n = 10), CM + 20 μM PD150606 (**ii**; n = 7), or DM (**iii**; n = 10). Scale bars, 20 μm. (**C**) Summary data, relative distribution of the Myc epitope signal in LPeD1. Asterisk, statistical significance (one-way ANOVA), *P* < 0.01–0.001.

Calpain-mediated menin cleavage is particularly intriguing considering our previous finding that LPeD1 neurons exhibit spontaneous activity-dependent calcium oscillations in response to NTF, which are required for the functional expression of excitatory nAChR^16^. We next generated a *MEN1* construct with 5′ (N-terminal) c-Myc (Myc) and 3′ (C-terminal) hemagglutinnin (HA) epitope tags (Fig. 3A), reasoning that calpain-dependent menin cleavage in response to NTF-induced calcium oscillations would result in separation of the epitope tags. Synthetic mRNA was microinjected into LPeD1 neurons cultured in DM, CM, or CM + 20 μM PD150606, and the subcellular distribution of Myc and HA epitopes was determined with ICC (Fig. 3B,C; n ≥ 7; see also Fig. S3, Table S2). In CM we observed separation of the Myc and HA signals, with the Myc epitope localized predominantly in the nucleus, and the HA eptiope localized predominantly in the cytoplasm. This separation reveals that the corresponding N-terminal menin fragment (N-menin) is maintained within neurons following proteolytic cleavage, and is specifically targeted to the nucleus. Calpain inhibition decreased nuclear Myc eptiope localization and increased cytoplasmic colocalization of Myc and HA epitopes, indicative of a shift towards full-length menin (*P* = 0.003, one-way ANOVA). In DM we observed predominantly cytoplasmic overlapping Myc and HA signals, indicative of full-length menin (*P* < 0.001). Thus, it appears that NTF signaling, in addition to activating the molecular signals for *MEN1* gene induction^9^, simultaneously provides the high concentration of intracellular calcium^16^ required for calpain activation and menin proteolytic cleavage. When taken together, our observations suggest that the differential targeting of menin, N-menin and C-menin within neurons may serve to mediate distinct functions during synaptogenesis.

### Postsynaptic recruitment of the C-menin fragment requires NTF- and activity-dependent signaling

**Figure 4:**
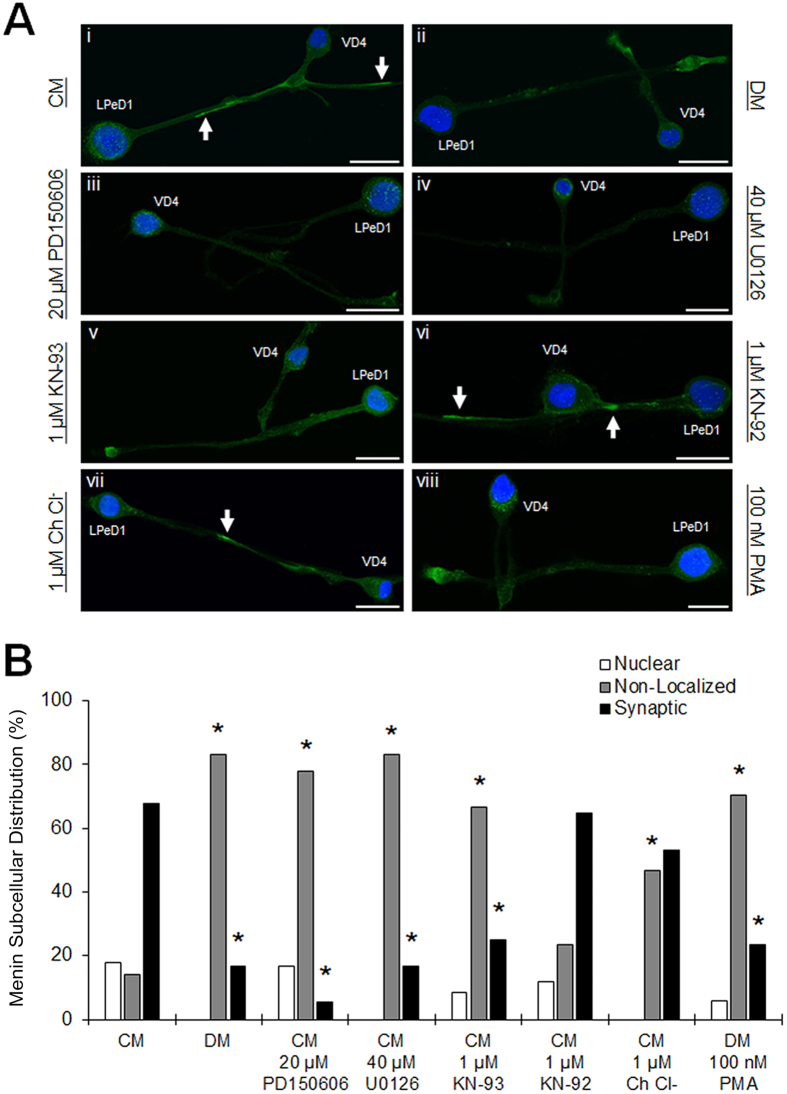
Postsynaptic recruitment of C-menin requires NTF- and activity-dependent signaling. (**A**) ICC of menin in axon-axon paired VD4-LPeD1 cultured in CM (**i**; NTF-rich; n = 28), DM (**ii**; NTF-poor; n = 12), CM + 20 μM PD150606 (**iii**; calpain inhibitor; n = 18), CM + 40 μM U0126 (**iv**; MAPK cascade inhibitor; n = 12), CM + 1 μM KN-93 (**v**; CaMKII inhibitor; n = 12), CM + 1 μM KN-92 (**vi**; inactive analogue; n = 17), CM + 1 μM Ch Cl^−^ (**vii**; PKC inhibitor; n = 15), or DM + 100 nM PMA (**viii**; PKC activator; n = 17). Arrows, synaptic sites. Scale bars, 50 μm. (**B**) Summary data, subcellular distribution of menin. Asterisks, statistical significance between distribution frequencies, relative to CM (Chi-squared test), *P* < 0.05–0.001.

We have characterized a variety of receptor tyrosine kinase- and activity-dependent mechanisms required for excitatory cholinergic synaptogenesis between VD4-LPeD1^9^,^16^,^17^,^18^. If excitatory synaptogenesis were dependent upon postsynaptic clustering of the C-menin fragment, we reasoned that synaptic recruitment would exhibit kinase-dependent regulation in alignment with some of these previously identified mechanisms. To this end, VD4-LPeD1 axon-axon pairs were cultured in the presence or absence of various kinase inhibitors and menin ICC was used to screen for candidate molecular pathways underlying the synaptic recruitment of C-menin (n ≥ 12 each). In VD4-LPeD1 cultured in CM, menin was mostly localized to the synapse (Fig. 4Ai; Table S3, CM data is repeated here from Fig. 1), whereas DM showed primarily non-localized menin (Fig. 4Aii; *P* = 0.003, Chi-squared test, synaptic distribution relative to CM reported in text). With calpain inhibition, neurons showed reduced synaptic and increased cytoplasmic menin (Fig. 4Aiii; CM + 20 μM PD150606; *P* < 0.001), which is consistent with our observations of menin and C-menin subcellular localizations (see Figs 2 and 3), and suggests that the synaptic α-menin signal specifically depicts the C-menin fragment. With disruption of the MAPK cascade downstream of receptor tyrosine kinase signaling, menin was primarily non-localized (Fig. 4Aiv; CM + 40 μM U0126; *P* = 0.003). With inhibition of the activity-dependent kinase CaMKII, menin fails to localize to the synapse (Fig. 4Av; CM + 1 μM KN-93; *P* = 0.013), but exhibits distribution patterns near identical to CM with the inactive analogue (Fig. 4Avi; CM + 1 μM KN-92; *P* = 0.828). Neither a PKC inhibitor (Fig. 4Avii; CM + 1 μM chelerythrine chloride (Ch Cl^−^); *P* = 0.348) nor a PKC activator (Fig. 4Aviii; DM + 100 nM phorbol 12-myristate 13-acetate (PMA); *P* = 0.004) affected the synaptic recruitment of menin. These observations indicate that phosphorylation of the C-menin fragment is likely required for synaptic targeting, and that this phenomenon is regulated by the same NTF-induced signaling mechanisms required for excitatory synaptogenesis between VD4-LPeD1 (references above).

### The C-menin fragment mediates postsynaptic consolidation of excitatory nAChR

**Figure 5:**
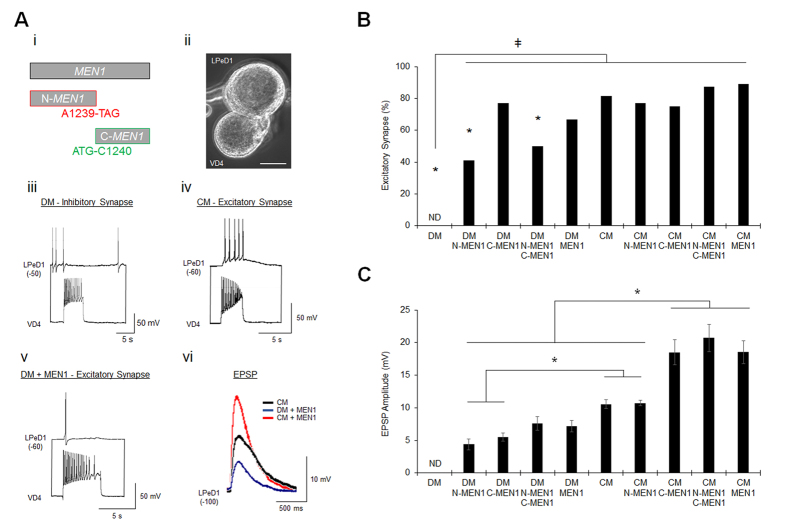
The C-menin fragment mediates postsynaptic consolidation of excitatory nAChR. (**A**) Schematic of *MEN1*, N-*MEN1*, and C-*MEN1* mRNA constructs (**i**). Soma-soma paired VD4-LPeD1 (**ii**). Scale bar, 20 μm. Inhibitory synapse in DM, VD4 activity elicits hyperpolarization in LPeD1 (**iii**). Excitatory synapse in CM, VD4 activity elicits depolarization in LPeD1 (**iv**). *MEN1* mRNA microinjection in LPeD1 induces an excitatory synapse in DM (**v**). Representative EPSP traces illustrate *MEN1*–induced (DM + *MEN1*) and *MEN1*-potentiated (CM + *MEN1*) EPSPs (**vi**). (**B**) Summary data, incidence of VD4-LPeD1 excitatory synapse formation in DM + H_2_O (n = 14), DM + N-*MEN1* (n = 17), DM + C-*MEN1* (n = 13), DM + N-*MEN1* + C-*MEN1* (n = 20), DM + *MEN1* (n = 15), CM + H_2_O (n = 43), CM + N-*MEN1* (n = 13), CM + C-*MEN1* (n = 12), CM + N-*MEN1* + C-*MEN1* (n = 8), and CM + *MEN1* (n = 9). mRNA was microinjected only into LPeD1 (see also Fig. S4). ND, excitatory synapses not detected. Asterisks, statistical significance relative to CM (Chi-squared test), *P* < 0.05–0.001. ǂ, statistical significance relative to DM, *P* < 0.05–0.001. (**C**) Summary data, mean EPSP amplitudes of VD4-LPeD1 synapses, as in (**B**). ND, EPSPs not detected. Error bars, SEM. Asterisks, statistical significance (one-way ANOVA), *P* < 0.05–0.001.

The evolutionary conservation of the menin ROI and calpain cleavage site suggests that there is a specific function for one or both of the fragments that is essential to the molecular actions of menin. To dissect the functional roles of N- and C-menin fragments in *MEN1*-dependent synaptogenesis, we generated 5′-truncated (C-*MEN1*) and 3′-truncated (N-*MEN1*) constructs at the end of the ROI adjacent to the calpain cleavage site (Fig. 5A). *MEN1* mRNA produces menin, N-*MEN1* mRNA produces the N-menin fragment, and C-*MEN1* mRNA produces the C-menin fragment. To characterize changes in synaptic physiology induced by the menin fragments, VD4-LPeD1 were paired in a soma-soma configuration in DM or CM, and LPeD1 was microinjected with synthetic *MEN1*, N-*MEN1*, C-*MEN1*, or N-*MEN1* + C-*MEN1* mRNA. We performed intracellular recordings to determine the incidence of excitatory synaptogenesis and measured the amplitudes of excitatory postsynaptic potentials (EPSPs) as an indication of synaptic strength (Fig. 5A–C; n ≥ 8; see also Fig. S4, Table S4). No excitatory synapses were observed in DM + H_2_O vehicle control (*P* < 0.001, Chi-squared test, relative to CM). LPeD1 mRNA microinjection of C-*MEN1* or *MEN1* in DM induced full rescue of the incidence of excitatory synaptogenesis (*P* > 0.05). mRNA microinjection of N-*MEN1* or N-*MEN1* + C-*MEN1* in DM induced partial rescue of excitatory synaptogenesis, as the incidence of excitatory synapses was significant relative to DM (*P* = 0.006, 0.002), but also CM (*P* = 0.002, 0.010). N-*MEN1* + C-*MEN1* or *MEN1* mRNA microinjection in DM induced full rescue of EPSP amplitudes (*P* > 0.05, one-way ANOVA), whereas EPSP amplitudes were reduced in N-*MEN1* or C-*MEN1* mRNA microinjected samples (*P* = 0.001,  < 0.001). These observations suggest, on the one hand, that there may be some redundancy in the function of menin, N-menin and C-menin in excitatory synaptogenesis, but on the other, that the independent molecular functions of the C-menin and N-menin fragments alone are insufficient for the full induction of excitatory synaptogenesis.

The observation that the C-menin fragment induced full synaptogenic rescue but only ~50% EPSP amplitudes supports a requirement for NTF-dependent C-menin synaptic recruitment in postsynaptic development (see Fig. 4). We therefore hypothesized that overexpression of the C-menin fragment, but not the N-menin fragment, in LPeD1 would potentiate excitatory synaptic transmission in the presence of NTF. The incidence of excitatory synapse formation between VD4-LPeD1 was unaffected by mRNA microinjection in CM (Fig. 5B; *P* > 0.05, Chi-squared test). Consistent with the above hypothesis, C-*MEN1*, N-*MEN1* + C-*MEN1*, or *MEN1* (which provides the C-menin ‘precursor protein’) mRNA microinjection induced ~2-fold synaptic potentiation (Fig. 5C; *P* = 0.044, 0.031, 0.033, one-way ANOVA), but N-*MEN1* mRNA microinjection did not (*P* = 1.000). These data support a role for C-menin synaptic recruitment in the postsynaptic consolidation of excitatory nAChR.

**Figure 6:**
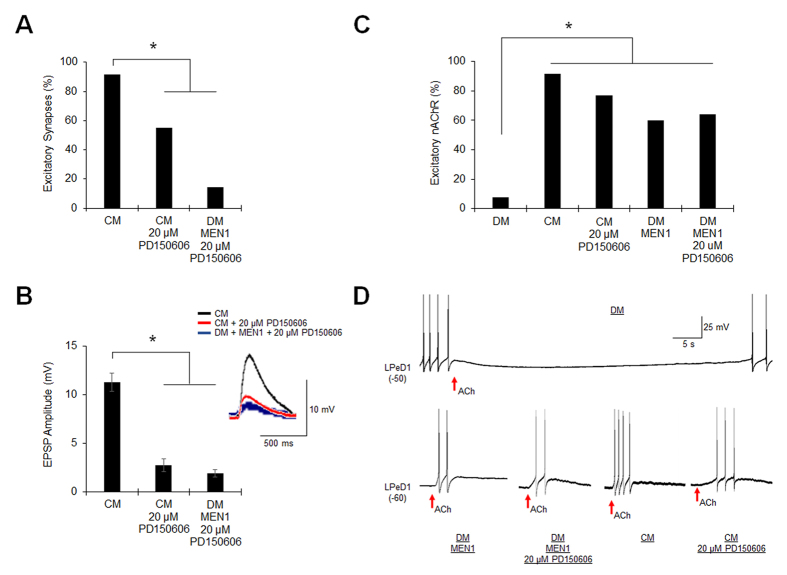
The C-menin fragment is required for postsynaptic consolidation but not functional expression of excitatory nAChR. (**A**) Incidence of VD4-LPeD1 excitatory synapse formation in CM + 0.1% DMSO (n = 12), CM + 20 μM PD150606 (n = 20), or DM + *MEN1* + 20 μM PD150606 (n = 14; mRNA was injected only into LPeD1). Asterisk, statistical significance relative to CM (Chi-squared test), *P* < 0.05–0.001. (**B**) Mean EPSP amplitudes, as in (**A**). Error bars, SEM. Insert shows representative EPSP traces. Asterisk, statistical significance (one-way ANOVA), *P* < 0.001. (**C**) Incidence of excitatory nAChR expression in single LPeD1 cultured in DM + H_2_O (n = 13), CM + 0.1% DMSO (n = 12), CM + 20 μM PD150606 (n = 13), DM + *MEN1* (n = 10), or DM + *MEN1* + 20 μM PD150606 (n = 14). Asterisk, statistical significance relative to DM (Chi-squared test), *P* < 0.01–0.001. (**D**) Representative traces, as in (**C**). ACh application (1 μM, arrow) in single LPeD1 above firing threshold (inhibitory nAChR, top) or below firing threshold (excitatory nAChR, bottom).

We next sought to determine whether excitatory synaptogenesis is dependent upon the generation of menin proteolytic fragments. While CM induces bursting activity in LPeD1, DM-cultured LPeD1 neurons also exhibit spontaneous activity^18^, which could be sufficient for low levels of menin proteolytic cleavage and underlie *MEN1*-induced excitatory synaptogenesis in the absence of NTF. To this end, intracellular recordings were made from VD4-LPeD1 pairs cultured in CM, CM + 20 μM PD150606, or DM + *MEN1* mRNA + 20 μM PD150606 (Fig. 6A,B; n ≥ 12; Table S5). Relative to CM, calpain inhibition in CM reduced excitatory synapse formation (*P* = 0.030, Chi-squared test) and also inhibited *MEN1*-induced excitatory synaptogenesis in DM (*P* < 0.001). Synaptic transmission was also reduced upon calpain inhibition in both CM and DM + *MEN1* mRNA (*P* < 0.001, one-way ANOVA). Recordings were next made from single LPeD1 neurons to determine whether the functional expression of excitatory nAChR in the postsynaptic neuron induced by *MEN1*^9^ is also contingent upon the generation of proteolytic fragments (Fig. 6C,D; n ≥ 10; Table S5). Single LPeD1 neurons cultured in DM exhibited an inhibitory response to exogenous ACh application, indicative of the functional expression of anionic nAChR. Single LPeD1 neurons cultured in CM exhibited an excitatory response to ACh (*P* < 0.001, Chi-squared test, relative to DM), indicative of the functional expression of cationic nAChR. DM + *MEN1* mRNA induced the expression of cationic nAChR in LPeD1 (*P* = 0.007). This nAChR anionic to cationic functional switch was not dependent upon the production of menin fragments, as an excitatory response to ACh was observed in LPeD1 cultured in CM + 20 μM PD150606 (*P* < 0.001), as well as DM + *MEN1* mRNA + 20 μM PD150606 (*P* = 0.002). Taken together, these data suggest that, while full length menin can induce the functional expression of excitatory nAChR, *MEN1*-dependent excitatory synaptogenesis requires generation of the C-menin fragment to mediate the postsynaptic recruitment of excitatory nAChR.

### Menin fragments coordinate subunit-specific transcriptional upregulation and synaptic targeting of excitatory nAChR

**Figure 7:**
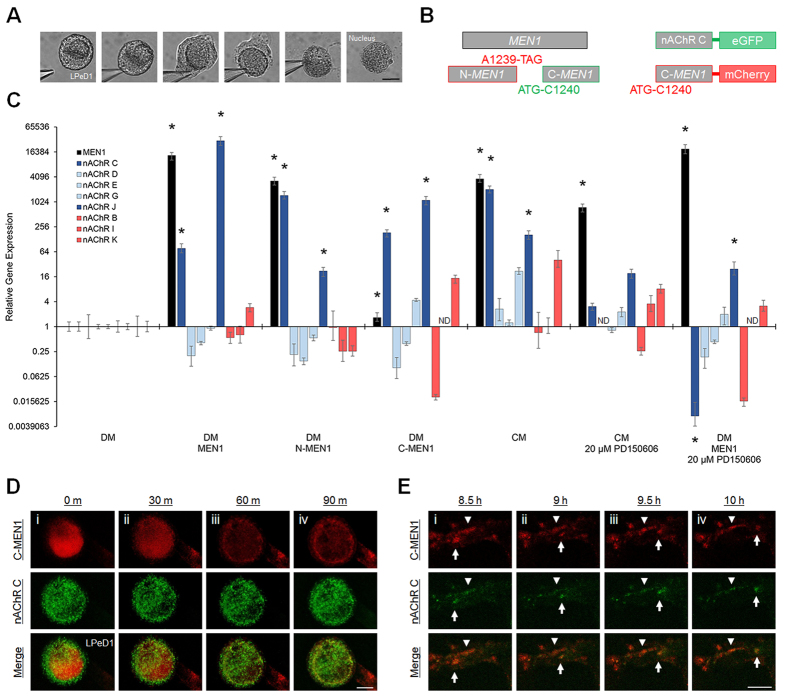
Menin fragments coordinate subunit-specific transcriptional upregulation and synaptic targeting of excitatory nAChR. (**A**) LPeD1 cytoplasm isolation for qPCR. Scale bar, 20 μm. (**B**) Schematic of mRNA constructs. (**C**) Relative gene expression in LPeD1 cultured in DM + *MEN1* (n = 2), DM + N-*MEN1* (n = 2), DM + C-*MEN1* (n = 2), CM + 0.1% DMSO (n = 3), CM + 20 μM PD150606 (n = 3), or DM + *MEN1* + 20 μM PD150606 (n = 2). Fold change gene expression is shown relative to expression levels in LPeD1 cultured in DM + H_2_O (n = 3). Each n represents a pooled sample of 12–15 single cells and 3 qPCR triplicate replicates. Dark blue bars depict excitatory nAChR C and J subunits upregulated by menin/fragments, light blue bars depict other excitatory nAChR subunits, red bars depict inhibitory nAChR subunits. ND, qPCR signal not detected. Error bars, SEM. Asterisks, statistical significance (pair wise fixed reallocation randomization test) for *MEN1*, nAChR C, and nAChR J, *P* < 0.05–0.001 (shown for clarity; other statistically significant differences were observed, see Table S6). (**D,E**) Live cell imaging of LPeD1 + C-*MEN1*-mCherry + nAChRC-eGFP, axon-axon paired with VD4, following the addition of CM (n = 8). Scale bars, 20 μm. (**D**) C-menin nuclear export at CM t = 0 m (**i**), 30 m (**ii**), 60 m (**iii**) and 90 m (**iv**). (**E**) Stable (arrowhead) and motile (arrow) C-menin and nAChR C puncta in the LPeD1 axon at CM t = 8.5 h (**i**), 9 h (**ii**), 9.5 h (**iii**) and 10 h (**iv**).

Considering our evidence for the nuclear localization of menin fragments (e.g. see Figs 1A, 3Bi and S4), we revisited our initial hypothesis that menin induces the transcriptional upregulation of excitatory nAChR subunits to prime postsynaptic neurons for excitatory synaptogenesis. To characterize the transcriptional influence of menin versus the proteolytic fragments on the expression of excitatory nAChR, the cytoplasm of LPeD1 neurons, cultured in DM and microinjected with *MEN1*, N-*MEN1* or C-*MEN1* mRNA, was isolated for single-cell qPCR (Fig. 7A–C). We also evaluated the expression of nAChR in LPeD1 cultured in CM to determine the profile of nAChR expression induced by NTF signaling. LPeD1 neurons cultured in DM + H_2_O served as a baseline to which the expression of nAChR in experimental samples was compared (n = 2–3 independent experiments each; n represents a pooled sample of 12–15 single cells and 3 qPCR triplicate replicates). We tested for all 12 *Lymnaea* nAChR subunits, and detected consistent qPCR signals for excitatory subunits C, D, E, G, J and inhibitory subunits B, I, K in LPeD1. nAChR F, H, L qPCR signals were observed infrequently, and nAChR A was not observed (data not shown). Relative to DM, *MEN1* mRNA microinjection induced the transcriptional upregulation of excitatory nAChR C and J subunits (Fig. 7C; *P* < 0.05–0.001, pair wise fixed reallocation randomization test; see Table S6). N-*MEN1* upregulated nAChR C and J subunits, and also induced the transcriptional upregulation of endogenous *MEN1* expression. C-*MEN1* upregulated nAChR C and J without inducing endogenous *MEN1* expression. CM induced the transcriptional upregulation of endogenous *MEN1* as well as excitatory nAChR C and J subunits, suggesting that NTF-induced transcription of excitatory nAChR subunits likely occurs via menin. CM and N-*MEN1* induced comparatively higher levels of nAChR C subunit expression, whereas *MEN1* and C-*MEN1* induced comparatively higher levels of nAChR J. This trend suggests that under appropriate physiological conditions (i.e. NTF stimulation and calpain activation), the N-menin fragment (i) amplifies *MEN1* expression through feed-forward autoregulation, and (ii) induces a specific expressional profile of cationic nAChR subunits to promote excitatory synaptogenesis. To determine whether generation of menin fragments is required for nAChR C expression, we also cultured LPeD1 neurons in CM + 20 μM PD150606 or DM + *MEN1* mRNA + 20 μM PD150606. In line with our hypothesis, full length menin resulting from calpain inhibition in either CM or DM was insufficient to induce transcriptional upregulation of nAChR C.

In light of the C-menin-induced synaptic potentiation that we observed (see Fig. 5C), we next sought to determine whether the excitatory nAChR C subunit upregulated by N-menin was subsequently targeted to synaptic sites with C-menin. To this end, we generated mCherry-tagged C-*MEN1* and eGFP-tagged nAChR C constructs, and synthetic mRNA was microinjected into LPeD1. Axon-axon paired VD4-LPeD1 neurons were maintained in DM for 8–24 h after mRNA microinjection, and live cell imaging was performed for ≥8 h following the addition of CM (n = 8). We observed nuclear localization of the C-menin fragment in 1/8 (13%) LPeD1 neurons, and the addition of CM induced nuclear export (Fig. 7D; see also Fig. S5). Along the LPeD1 axon, we observed both stable and motile puncta of C-menin co-localized with nAChR C in 5/8 (63%) VD4-LPeD1 pairs (Fig. 7E). These data parallel the synaptic and nuclear distribution patterns observed with α-menin ICC (see Fig. 4).

**Figure 8:**
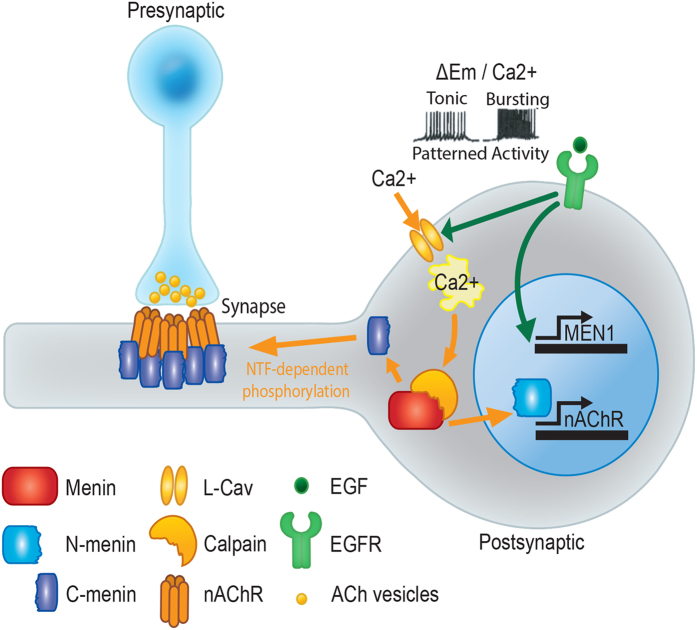
A model for the coordination of nuclear transcription and postsynaptic clustering of excitatory nAChR by menin proteolytic fragments.

Taken together, our data support a model in which (i) NTF-induced activity promotes calpain activation and proteolytic cleavage of menin; (ii) nuclear targeting of the N-menin fragment further amplifies *MEN1* expression and mediates transcriptional upregulation of the nAChR C subunit, which may be required for the synaptic targeting of excitatory nAChR; and (iii) NTF-induced phosphorylation promotes postsynaptic targeting of the C-menin fragment, which is required for the clustering of excitatory nAChR (Fig. 8).

## Discussion

While previous studies on the role of menin in CNS synapse formation and plasticity have characterized its functional significance, these have so far been unable to delineate the underlying molecular mechanisms. Our report provides the first evidence for a cytoplasmic function of menin, which was previously thought to be primarily a nuclear protein, in the postsynaptic targeting of excitatory nAChR. This study is also the first to demonstrate that menin influences subunit-specific transcription of nAChR in neurons. Here, we propose a novel model for excitatory synaptogenesis in which the nuclear transcription and postsynaptic targeting of neurotransmitter receptors is coordinated via differential localization and distinct molecular functions of two proteolytic fragments of a single gene product.

### Proteolytic Cleavage

NTF-mediated molecular signaling events influence nearly all aspects of the development and function of neuronal circuits, from neurogenesis and proliferation to neuronal outgrowth, synapse formation, maturation and plasticity^19^. NTFs are well known to induce distinct patterns of neuronal activity necessary for synapse formation^20^. In *Lymnaea* LPeD1 neurons, we have previously shown that CM induces stereotypical activity patterns and calcium oscillations required for the conversion from inhibitory to excitatory nAChR^16^,^18^. Calpains, which are ubiquitously expressed, are one of many classes of effector proteins underlying the signal transduction of calcium as a second messenger, and calpain-dependent proteolysis has been implicated in a diverse array of calcium-dependent cellular processes, including apoptosis, adhesion, cytoskeletal reorganization, synaptic plasticity, and neurodegeneration^21^. A number of tumor suppressor proteins have been found to be cleaved by calpain, including p53^22^, NF2^23^, PTEN^24^, as well as the *Dlg* homolog PSD-95^25^. In the present study, we have shown that proteolytic cleavage of menin in neurons allows NTF-dependent molecular signaling to coordinate nuclear and cytoplasmic events prerequisite for excitatory synaptogenesis. Our observations also support a general model in which the production of proteolytic fragments may play a common role in the regulation of cell-cell interactions by tumor suppressors.

### Transcriptional Regulation

Synapse formation and long-lasting forms of synaptic plasticity, involving the growth of new synaptic connections, require activity-dependent gene induction, *de novo* protein synthesis, and site-specific targeting of effector proteins^26^. The cyclic AMP response element binding protein (CREB) cascade is the prototypical mechanism through which extracellular stimuli are transduced into gene expression changes necessary for synaptic development and remodeling^27^,^28^,^29^,^30^,^31^. The transcription factor CREB is a convergence point for multiple second messenger systems, allowing neurotransmitter-receptor interactions, calcium influx, and impulse activity to cooperatively influence activity-dependent gene transcription^32^,^33^. While there is currently no evidence for the transcriptional activation of *MEN1* by CREB, five *cis*-regulatory regions have been identified in the promoter region of *MEN1*, and the activity of these regulatory elements is dependent upon cellular context (endocrine vs. non-endocrine cell type)^34^, indicating that this facilitates cell type-specific transcriptional regulation. This could conceivably be extended to explain the responsiveness of *MEN1* gene expression to context-dependent molecular signals in neurons, such as NTF-activated second messenger systems. For instance, calcium influx through L-type calcium channels (L-Cav) is critically involved in the activation of immediate early genes, typically transcription factors, that mediate subsequent transcriptional changes underlying synapse formation and plasticity^35^. L-Cav signal transduction to the nucleus is mediated via locally aggregated CaMKII^36^, which has also been show to activate CREB^32^. We have previously reported that inhibition of L-Cav prevents the expression of excitatory nAChR in *Lymnaea* LPeD1 neurons^16^, and that this can be bypassed by the injection of *MEN1* mRNA^9^. Considering the findings of the present study, these observations suggest that menin is the molecular intermediary between NTF-induced activity-dependent signaling and the transcriptional upregulation of excitatory nAChR subunits relevant to excitatory synaptogenesis.

Menin binds to both transcriptional activators (e.g. MLL1, SMADs) and repressors (e.g. JunD, NFκβ) to regulate gene transcription in response to numerous cell signaling cascades^4^. Menin is also known to bind directly to DNA and influence gene transcription^37^. Furthermore, the transcription of *MEN1* is influenced by the intracellular levels of menin, indicating a promoter system that facilitates auto-regulation^34^. This provides support for our observation that the N-menin fragment induced endogenous *MEN1* expression. The transcriptional influence of menin and the C-terminal fragment, without nuclear localization, could occur indirectly through the regulation of other transcription factors. For instance, the nuclear export of menin has been reported to reduce nuclear accumulation of β-catenin and thereby influence its transcriptional activity^7^. Considering that full length menin was localized primarily to the cytoplasm, and that addition of NTF induced the export of nuclear localized C-menin, our observations suggest that the transcriptional influence of menin in *Lymnaea* neurons depends primarily upon the nuclear localized N-menin fragment.

Neuronal nAChR are diverse and heterogeneous due to the variety of possible subunit combinations that can comprise the pentameric channels, and the factors that regulate specific patterns of expression, transcriptional regulation, assembly and trafficking are not well understood^38^. Here, we identified *MEN1-*induced transcriptional upregulation of excitatory nAChR C and J subunits in *Lymnaea* LPeD1 neurons, where nAChR C subunit expression was specifically promoted by the N-menin fragment. As nAChR C (α-type subunit) and J (β-type subunit) are not known to form functional homopentamers^39^, these are likely to be accessory subunits that modulate the assembly, function, or trafficking of nAChR. Here, we show that calpain inhibition both prevented nAChR C upregulation and excitatory synapse formation between VD4-LPeD1, but not nAChR J upregulation or excitatory nAChR expression in single LPeD1 neurons. Taken together, these findings suggest that NTF stimulation induces the transcriptional upregulation of the nAChR C subunit via N-menin, and raise the possibility that nAChR C expression may be required for the formation of nAChR channels that are competent for synaptic targeting.

### Postsynaptic Recruitment

In this study, we describe for the first time the functional significance of the C-menin proteolytic fragment and its NTF-dependent postsynaptic targeting. Phosphorylation of human menin at C-terminal residues Ser^543^ and Ser^583^ has been identified, and this was found to have no effect on nuclear localization or transcriptional regulation^40^. The phosphorylation of these C-terminal serine residues and the absence of a transcriptional effect provides support for our observation that the postsynaptic recruitment of C-menin requires phosphorylation by the Ser/Thr kinases MAPK or CaMKII. As the corresponding serine residues are conserved in *Lymnaea* menin, we suspect that this may serve as the molecular signal for synaptic localization of the C-menin fragment.

The synaptic potentiation induced by C-menin and its co-localization with nAChR is analogous to the effects described for the postsynaptic clustering of glutamate receptors by the molecular scaffold PSD-95 in mammalian neurons^41^. Menin is also known to act as a molecular scaffold, mediating crosstalk between multiple signaling pathways in the regulation of gene transcription^4^. If this scaffolding function is maintained in the C-menin fragment, our data would raise the intriguing possibility that the C-menin fragment is the previously unidentified molecular scaffold for nAChR clustering in neurons. Cholinergic synaptic development at the neuromuscular junction (NMJ) is dependent upon agrin signaling through muscle-specific kinase (MuSK) receptors to mediate the clustering of nAChR via the intracellular effector rapsyn^42^. While agrin, MuSK, and rapsyn are expressed in neurons and have been found to influence the function of cholinergic synapses in the CNS^43^,^44^, rapsyn is not essential for postsynaptic nAChR clustering at central synapses^45^. By contrast, we have previously demonstrated that postsynaptic *MEN1* knockdown inhibits NTF-dependent excitatory cholinergic synaptogenesis^8^, and that *MEN1* induces excitatory cholinergic postsynaptic development in the absence of NTF signaling^9^. When taken together, our observations illustrate both the necessity and sufficiency for *MEN1* in excitatory cholinergic synaptogenesis between *Lymnaea* central neurons. In murine models, independent reports have shown that peripheral nerve injury upregulates both *MEN1*^10^,^11^,^12^ and the modulatory nAChR α5 subunit^46^ in the spinal cord dorsal horn to produce neuropathic pain. While the induction of neuropathic pain by menin has been characterized in terms of glutamatergic hyperexcitability^12^, this could also be consistent with *MEN1-*induced cholinergic plasticity resulting in glutamatergic facilitation^47^, as cholinergic innervations are present in the dorsal horn^48^. If the molecular actions of menin in neurons have indeed been conserved across evolution, this may be a *MEN1*-dependent transcriptional upregulation and synaptic clustering of α5-containing nAChR, in response to enhanced NTF signaling following injury^49^.

Taken together, our observations on the role of calpain-dependent menin cleavage in excitatory synapse formation demonstrate for the first time the molecular actions of menin in neurons, and also reveal a novel synaptogenic mechanism in which a single gene product coordinates the nuclear transcription and postsynaptic targeting of neurotransmitter receptors. Unraveling the distinct molecular functions of the menin proteolytic fragments and whether these are tissue specific or context dependent may ultimately provide new insights into menin’s actions as a tumor suppressor, and further fundamental knowledge regarding the development of cholinergic synapses in the CNS, as well as neurodegenerative conditions such as Alzheimer’s disease in which NTF^50^, nAChR^51^, and tumor suppressor function^52^,^53^ are compromised.

## Methods

### Animals and Neuronal Cell Culture

*Lymnaea stagnalis* were raised under standard conditions in freshwater aquaria at room temperature (RT; ~22 °C), and fed a diet of lettuce. Animals (6–8 weeks old for cell culture; >3 months old for protein samples and CM) were sacrificed by removal of the CNS after anesthesia was induced with a 10% Listerine solution (10 m). Identified neurons were isolated from trypsinized CNS by suction applied through a glass pipette and plated on poly-L-lysine coated glass culture dishes, as previously described in detail^54^,^55^. Isolated neurons were maintained overnight (15–20 h) in defined media (DM; trophic factor-deficient media; L-15; Life technologies; special order) or CNS conditioned DM (CM; trophic factor-rich media).

### Molecular Biology

We have recently described the cloning of *Lymnaea-MEN1*^9^. Myc (5′) and HA (3′) epitope tags were introduced onto this construct using Myc and HA-encoding primers with the KAPA HiFi HotStart ReadyMix PCR kit (Kapa Biosystems). For N-*MEN1* (menin M^1^- A^413^) the stop codon TAG was introduced after A1239. For C-*MEN1* (menin Q^414^-V^759^) the start codon ATG was introduced ahead of C1240. nAChR C lacking the terminal stop codon was cloned from *Lymnaea* CNS cDNA, generated as previously described^9^. eGFP was cloned from the pWPI lentiviral construct (Addgene) and inserted in frame downstream of nAChR C. mCherry was cloned from the pSicoR-Ef1a-mCh lentiviral vector (Addgene) and extended onto the C-*MEN1* sequence by site-overlap-extension PCR, which eliminated the C-*MEN1* stop codon. Primers are shown in Table S7. Constructs were inserted into pBlueScript SK- (Clontech). Synthetic mRNA was made using mMESSAGE mMACHINE T7 Ultra Transcription kit (Ambion). Kits were used according to manufacturers’ instructions. mRNA was microinjected into LPeD1 neurons with a low resistance glass electrode, as described previously^9^. Molecular-grade water was microinjected as a vehicle control. Sequence alignments were generated with Clustal Omega (EMBL-EBI).

Quantitative (q)PCR expression profiling of LPeD1 neurons was performed as we have recently described in detail^9^. Gene specific primers are shown in Tables S8 and S9. Efficiency values for qPCR primers ranged between 90–110% (R^2^ = 0.96–0.99). Negative controls and validations were as previously described^9^. Changes in gene expression (relative to LPeD1 + H_2_O DM, normalized to 18s rRNA and β-tubulin), and statistical significance was determined using REST-2009^56^.

### Immunocytochemistry and Microscopy

Neurons were fixed 30 m with 4% paraformaldehyde and 0.2% picric acid (Sigma-Aldrich) in 1 × PBS, and permeabilized for 1 h with incubation media (IM) containing 0.2% Triton, 5% fetal calf serum, and 0.25% fish gelatin in 1 × TBS. Primary antibodies (α-menin [Bethyl Laboratories, A300-105A]; α-5-HT [AbCam, ab16007]; α-FMRFamide^57^; α-hemagglutinnin [Covance, MMS-101P], α-c-Myc [Sigma-Aldrich, C3956]) were used at 1:500 in IM for 1 h. Secondary antibodies [Alexa Fluor 488, 546 or 633 (Invitrogen)] were used at 1:100 in IM for 1 h. Three 15 m washes in 1 × PBS were performed after each incubation, and all incubations were performed at RT. Presynaptic terminals were labeled with 10 μM FM 1-43 (Molecular Probes) by 10 × 5s bursts induced in VD4 by current injection through a sharp electrode, then washed on ice with cold 1 × PBS and fixed as above. Neurons were mounted with MOWIOL containing DAPI. For live cell imaging, LPeD1 neurons were microinjected with C-*MEN1*-mCherry and nAChR C-eGFP mRNA, and VD4-LPeD1 axon pairs were maintained in DM for 8–24 h. Images were acquired at 10 m intervals for ≥8 h following the addition of CM. Confocal Z-stacks (1 μm) images were acquired using an A1R MP microscope (Nikon) under a CFI Plan Fluor 20x/0.75 MI objective (Nikon), with motorized positioning systems (Prior Scientific). Fluorophores were excited with 402, 488, 561 and 651 laser wavelengths in series and emissions collected through 450/50, 525/50, 595/50 and 700/75 filter cubes. Imaging parameters were kept the same amongst relevant samples. Images were acquired with NIS Elements v4.13.00 software (Nikon) and processed with ImageJ (NIH).

### Preparation of Protein Samples and Western Blotting

*Lymnaea* CNS tissue for protein samples was acutely dissected (25 CNS) or maintained in organ culture (3 CNS, 3 mL DM at RT for 72 h). Tissue was immediately frozen on dry ice and stored at −80 °C. CNS tissue was homogenized in lysis buffer containing 1% Triton, 50 mM Tris-HCl, 150 mM NaCl (pH 8.0) and a cOmplete Mini EDTA-free protease inhibitor cocktail tablet (Roche). Homogenate was incubated on a shaker for 2 h at 4 °C, centrifuged at 1,400 × g for 20 m at 4 °C and stored at −80 °C. Our protocol for subcellular fractionation was adapted from previously described methods^58^,^59^, with minor modifications. 150 *Lymnaea* CNS were homogenized by 20 strokes with a loose pestle followed by 10 strokes with a tight pestle in a Dounce homogenizer in homogenization buffer (HB; 0.3 M sucrose, 25 mM Tris-HCl [pH 7.4]). All buffers contained cOmplete Mini EDTA-free protease inhibitor cocktail (Roche) or Halt protease inhibitor cocktail (Thermo Scientific), and all steps were performed on ice or at 4 °C. Homogenate was centrifuged at 1,000 × g for 5 m to pellet crude nuclei (S1, P1). The pellet (P1) was resuspended in HB and centrifuged again at 1,000 × g for 5 m (S2, P2). Nuclear Fraction: the crude nuclei pellet (P2) was briefly homogenized by 10 strokes with a tight pestle in a Dounce homogenizer, then twice washed with N-buffer (10 mM NaCl, 3 mM MgCl_2_, 10 mM Tris-HCl [pH 7.4], 0.1% Nonidet-P40, 0.05% sodium deoxycholate, 10 mM N-ethyl malemide and 0.5% β-mercaptoethanol), and centrifuged at 1,000 × g for 5 m. The pellet was centrifuged through a 1.8 M sucrose cushion in 10 mM Tris-HCl (pH 7.4) at 30,000 × g for 30 m, then washed again with N-buffer and centrifuged at 1,000 × g for 5 m. The pellet was resuspended in RIPA buffer (150 mM NaCl, 10 mM Tris-HCl [pH 7.4], 1% Nonidet-P40, 1% sodium deoxycholate, 0.1% SDS, and 10 mM N-ethyl malemide), sonicated briefly, and centrifuged at 12,000 × g for 10 m to pellet debris. Cytoplasmic Fraction: supernatants (S1, S2) were combined and centrifuged at 17,000 × g for 30 m to pellet crude membranes (P3). The resulting supernatant was the soluble cytoplasmic fraction. Synaptic Fraction: The crude membrane pellet (P3) was resuspended in 1.2 M sucrose in 10 mM Tris-HCl (pH 7.4) and allowed to stand on ice for 30 m, then separated on a discontinuous sucrose density gradient established with 1.2, 0.8 and 0.3 M sucrose in 10 mM Tris-HCl (pH 7.4). This was ultracentrifuged for 30 m at 100,000 × g using a SW41 Ti rotor (Beckman). The interface between 1.2 and 0.8 M sucrose was collected and pelleted at 150,000 × g for 1 h using a TLS-55 rotor (Beckman). The pellet was resuspended in HB. Fractions were aliquoted and stored at −80 °C. Protein extracts (40 μg/lane) were resolved on SDS-PAGE gels and transferred to PVDF membranes (BioRad). Membrane blocking and antibody incubations (α-menin at 1:2000; α-β-actin [AbCam, ab8227] at 1:20000; α-β-tubulin [Sigma-Aldrich, T0198] at 1:2000; α-complexin [Synaptic Systems, 122 102] at 1:2000; α-histone H3 [Millipore, 06-599] at 1:2000) were performed with 5% skim milk powder + 0.1% Tween-20 in 1 × PBS, overnight at 4 °C or for 1 h at RT. Bands were detected with IRDye-800CW conjugated α-rabbit or α-mouse IgG (Li-Cor Biosciences) at 1:5000 for 1 h at RT. Three 15 m washes in 1 × PBS + 0.1% Tween-20 at RT were performed after each antibody incubation. Membranes were visualized using a Li-Cor Odyssey infra-red imager and bands were quantified using Odyssey v3.0 software.

### Electrophysiology

Intracellular current clamp recordings were used to characterize single cell physiology or *in vitro* synapses, as previously described in detail^16^. For single cell recordings ACh was dissolved into DM (1 μM), and applied using pressure application through a microelectrode (tip opening ~1–5 μm; 250 ms pulse, 10 PSI). An excitatory synapse was confirmed by depolarization of LPeD1 at −60 mV. The mean amplitude of 5 consecutive EPSPs at −100 mV was used as a measure of synaptic efficacy.

### Chemicals

Acetylcholine (ACh), PMA and Chelerythrine Cl^−^ were purchased from Sigma-Aldrich. PD150606 was purchased from Tocris. KN-92 and KN-93 were purchased from EMD Millipore. U0126 was purchased from Promega. Drugs were dissolved into DMSO, and 0.1% DMSO vehicle controls were performed for all experiments.

### Experimental Design and Statistical Analysis

To ensure reliability and replicability of the results, all reported data are derived from ≥2 independent experiments, conducted with tissue preparations from independent dissection sessions or cells from independent culture sessions. Sample sizes were limited by uncontrollable factors inherent to the cell culturing techniques used in this study. Data analysis, including fluorescence intensity measurements, subcellular distribution incidence, and electrophysiology, was performed blinded by acquisition file number.

Statistical analyses were performed using SPSS Statistics v22. Data distribution was assessed with Shapiro-Wilk test, and all data sets were normally distributed (*P* > 0.05), with the exception of two (*P* < 0.05), DM + N-MEN1 + C-MEN1 (Fig. 5C) and CM + 20 μM PD150606 (Fig. 6B). The non-normal distribution in these two data sets results from a bimodal distribution, reflecting preparations in which synapse formation either was or was not effectively induced/inhibited. Differences between two data sets were analyzed with Student’s independent samples T-test (2-sided), and differences amongst three or more data sets were analyzed with univariate ANOVA, with Tukey’s HSD *post hoc* test if variances were equal (Levene’s statistic *P* > 0.05) or Games-Howell *post hoc* test if variances were unequal (Levene’s statistic *P* < 0.05). Incidence data were assessed with Pearson’s Chi-squared test (2-sided). Differences in relative gene expression were determined via pair wise fixed reallocation randomization test using REST-2009.

## Additional Information

**How to cite this article**: Getz, A. M. *et al*. Two proteolytic fragments of menin coordinate the nuclear transcription and postsynaptic clustering of neurotransmitter receptors during synaptogenesis between *Lymnaea* neurons. *Sci. Rep.* **6**, 31779; doi: 10.1038/srep31779 (2016).

## Supplementary Material

Supplementary Information

## References

[b1] Chandrasekharappa S. C. . Positional cloning of the gene for multiple endocrine neoplasia-type 1. Science 276, 404–407 (1997). 9103196 10.1126/science.276.5311.404

[b2] Guru S. C. . Menin, the product of the MEN1 gene, is a nuclear protein. Proceedings of the National Academy of Sciences of the United States of America 95, 1630–1634 (1998). 9465067 10.1073/pnas.95.4.1630 PMC19125

[b3] Huang J. . The same pocket in menin binds both MLL and JUND but has opposite effects on transcription. Nature 482, 542–546, 10.1038/nature10806 (2012). 22327296 PMC3983792

[b4] Matkar S., Thiel A. & Hua X. Menin: a scaffold protein that controls gene expression and cell signaling. Trends in biochemical sciences 38, 394–402, 10.1016/j.tibs.2013.05.005 (2013). 23850066 PMC3741089

[b5] Green D. R. & Kroemer G. Cytoplasmic functions of the tumour suppressor p53. Nature 458, 1127–1130, 10.1038/nature07986 (2009). 19407794 PMC2814168

[b6] Budnik V. . Regulation of synapse structure and function by the Drosophila tumor suppressor gene dlg. Neuron 17, 627–640 (1996). 8893021 10.1016/s0896-6273(00)80196-8 PMC4661176

[b7] Cao Y. . Nuclear-cytoplasmic shuttling of menin regulates nuclear translocation of {beta}-catenin. Molecular and cellular biology 29, 5477–5487, 10.1128/MCB.00335-09 (2009). 19651895 PMC2756891

[b8] van Kesteren R. E. . Synapse formation between central neurons requires postsynaptic expression of the MEN1 tumor suppressor gene. The Journal of neuroscience: the official journal of the Society for Neuroscience 21, RC161 (2001). 11473132 10.1523/JNEUROSCI.21-16-j0004.2001 PMC6763132

[b9] Flynn N., Getz A., Visser F., Janes T. A. & Syed N. I. Menin: A Tumor Suppressor That Mediates Postsynaptic Receptor Expression and Synaptogenesis between Central Neurons of Lymnaea stagnalis. PloS one 9, e111103, 10.1371/journal.pone.0111103 (2014). 25347295 PMC4210270

[b10] Zhang X., Chen G., Xue Q. & Yu B. Early changes of beta-Catenins and Menins in spinal cord dorsal horn after peripheral nerve injury. Cellular and molecular neurobiology 30, 885–890, 10.1007/s10571-010-9517-9 (2010). 20369282 PMC11498821

[b11] Xu S. . Tumor suppressor menin mediates peripheral nerve injury-induced neuropathic pain through potentiating synaptic plasticity. Neuroscience 223, 473–485, 10.1016/j.neuroscience.2012.07.036 (2012). 22858595

[b12] Shen X. . Menin regulates spinal glutamate-GABA balance through GAD65 contributing to neuropathic pain. Pharmacological reports: PR 66, 49–55, 10.1016/j.pharep.2013.06.005 (2014). 24905306

[b13] Stewart C. . Characterization of the mouse Men1 gene and its expression during development. Oncogene 17, 2485–2493, 10.1038/sj.onc.1202164 (1998). 9824159

[b14] van Nierop P. . Identification of molluscan nicotinic acetylcholine receptor (nAChR) subunits involved in formation of cation- and anion-selective nAChRs. The Journal of neuroscience: the official journal of the Society for Neuroscience 25, 10617–10626, 10.1523/JNEUROSCI.2015-05.2005 (2005). 16291934 PMC6725845

[b15] DuVerle D. A., Ono Y., Sorimachi H. & Mamitsuka H. Calpain cleavage prediction using multiple kernel learning. PloS one 6, e19035, 10.1371/journal.pone.0019035 (2011). 21559271 PMC3086883

[b16] Xu F., Hennessy D. A., Lee T. K. & Syed N. I. Trophic factor-induced intracellular calcium oscillations are required for the expression of postsynaptic acetylcholine receptors during synapse formation between Lymnaea neurons. The Journal of neuroscience: the official journal of the Society for Neuroscience 29, 2167–2176, 10.1523/JNEUROSCI.4682-08.2009 (2009). 19228969 PMC6666333

[b17] Woodin M. A., Munno D. W. & Syed N. I. Trophic factor-induced excitatory synaptogenesis involves postsynaptic modulation of nicotinic acetylcholine receptors. The Journal of neuroscience: the official journal of the Society for Neuroscience 22, 505–514 (2002). 11784796 10.1523/JNEUROSCI.22-02-00505.2002 PMC6758671

[b18] Luk C. C. . Trophic factor-induced activity ‘signature’ regulates the functional expression of postsynaptic excitatory acetylcholine receptors required for synaptogenesis. Scientific reports 5, 9523, 10.1038/srep09523 (2015). 25827640 PMC4381329

[b19] Park H. & Poo M. M. Neurotrophin regulation of neural circuit development and function. Nature reviews. Neuroscience 14, 7–23, 10.1038/nrn3379 (2013). 23254191

[b20] Blum R., Kafitz K. W. & Konnerth A. Neurotrophin-evoked depolarization requires the sodium channel Na(V)1.9. Nature 419, 687–693, 10.1038/nature01085 (2002). 12384689

[b21] Wu H. Y. & Lynch D. R. Calpain and synaptic function. Molecular neurobiology 33, 215–236, 10.1385/MN:33:3:215 (2006). 16954597

[b22] Pariat M. . Proteolysis by calpains: a possible contribution to degradation of p53. Molecular and cellular biology 17, 2806–2815 (1997). 9111352 10.1128/mcb.17.5.2806 PMC232132

[b23] Kimura Y. . The involvement of calpain-dependent proteolysis of the tumor suppressor NF2 (merlin) in schwannomas and meningiomas. Nature medicine 4, 915–922 (1998). 10.1038/nm0898-915 9701243

[b24] Briz V. . Calpain-2-mediated PTEN degradation contributes to BDNF-induced stimulation of dendritic protein synthesis. The Journal of neuroscience: the official journal of the Society for Neuroscience 33, 4317–4328, 10.1523/JNEUROSCI.4907-12.2013 (2013). 23467348 PMC3657575

[b25] Lu X., Rong Y. & Baudry M. Calpain-mediated degradation of PSD-95 in developing and adult rat brain. Neuroscience letters 286, 149–153 (2000). 10825658 10.1016/s0304-3940(00)01101-0

[b26] Cohen S. & Greenberg M. E. Communication between the synapse and the nucleus in neuronal development, plasticity, and disease. Annual review of cell and developmental biology 24, 183–209, 10.1146/annurev.cellbio.24.110707.175235 (2008). PMC2709812 18616423

[b27] Bourtchuladze R. . Deficient long-term memory in mice with a targeted mutation of the cAMP-responsive element-binding protein. Cell 79, 59–68 (1994). 7923378 10.1016/0092-8674(94)90400-6

[b28] Casadio A. . A transient, neuron-wide form of CREB-mediated long-term facilitation can be stabilized at specific synapses by local protein synthesis. Cell 99, 221–237 (1999). 10535740 10.1016/s0092-8674(00)81653-0

[b29] Sadamoto H. . CREB in the pond snail Lymnaea stagnalis: cloning, gene expression, and function in identifiable neurons of the central nervous system. Journal of neurobiology 58, 455–466, 10.1002/neu.10296 (2004). 14978723

[b30] Dash P. K., Hochner B. & Kandel E. R. Injection of the cAMP-responsive element into the nucleus of Aplysia sensory neurons blocks long-term facilitation. Nature 345, 718–721, 10.1038/345718a0 (1990). 2141668

[b31] Yin J. C. . Induction of a dominant negative CREB transgene specifically blocks long-term memory in Drosophila. Cell 79, 49–58 (1994). 7923376 10.1016/0092-8674(94)90399-9

[b32] Dash P. K., Karl K. A., Colicos M. A., Prywes R. & Kandel E. R. cAMP response element-binding protein is activated by Ca2+/calmodulin- as well as cAMP-dependent protein kinase. Proceedings of the National Academy of Sciences of the United States of America 88, 5061–5065 (1991). 1647024 10.1073/pnas.88.11.5061 PMC51807

[b33] Deisseroth K., Bito H. & Tsien R. W. Signaling from synapse to nucleus: postsynaptic CREB phosphorylation during multiple forms of hippocampal synaptic plasticity. Neuron 16, 89–101 (1996). 8562094 10.1016/s0896-6273(00)80026-4

[b34] Fromaget M. . Functional characterization of a promoter region in the human MEN1 tumor suppressor gene. Journal of molecular biology 333, 87–102 (2003). 14516745 10.1016/j.jmb.2003.08.001

[b35] Murphy T. H., Worley P. F. & Baraban J. M. L-type voltage-sensitive calcium channels mediate synaptic activation of immediate early genes. Neuron 7, 625–635 (1991). 1657056 10.1016/0896-6273(91)90375-a

[b36] Wheeler D. G. . Ca(V)1 and Ca(V)2 channels engage distinct modes of Ca(2+) signaling to control CREB-dependent gene expression. Cell 149, 1112–1124, 10.1016/j.cell.2012.03.041 (2012). 22632974 PMC3654514

[b37] La P. . Direct binding of DNA by tumor suppressor menin. The Journal of biological chemistry 279, 49045–49054, 10.1074/jbc.M409358200 (2004). 15331604 PMC2858586

[b38] Colombo S. F., Mazzo F., Pistillo F. & Gotti C. Biogenesis, trafficking and up-regulation of nicotinic ACh receptors. Biochemical pharmacology 86, 1063–1073, 10.1016/j.bcp.2013.06.023 (2013). 23830821

[b39] van Nierop P. . Identification and functional expression of a family of nicotinic acetylcholine receptor subunits in the central nervous system of the mollusc Lymnaea stagnalis. The Journal of biological chemistry 281, 1680–1691, 10.1074/jbc.M508571200 (2006). 16286458

[b40] MacConaill L. E., Hughes C. M., Rozenblatt-Rosen O., Nannepaga S. & Meyerson M. Phosphorylation of the menin tumor suppressor protein on serine 543 and serine 583. Molecular cancer research: MCR 4, 793–801, 10.1158/1541-7786.MCR-06-0123 (2006). 17050672

[b41] El-Husseini A. E., Schnell E., Chetkovich D. M., Nicoll R. A. & Bredt D. S. PSD-95 involvement in maturation of excitatory synapses. Science 290, 1364–1368 (2000). 11082065

[b42] Gautam M. . Failure of postsynaptic specialization to develop at neuromuscular junctions of rapsyn-deficient mice. Nature 377, 232–236, 10.1038/377232a0 (1995). 7675108

[b43] Bose C. M. . Agrin controls synaptic differentiation in hippocampal neurons. The Journal of neuroscience: the official journal of the Society for Neuroscience 20, 9086–9095 (2000). 11124985 10.1523/JNEUROSCI.20-24-09086.2000 PMC6773041

[b44] Garcia-Osta A. . MuSK expressed in the brain mediates cholinergic responses, synaptic plasticity, and memory formation. The Journal of neuroscience: the official journal of the Society for Neuroscience 26, 7919–7932, 10.1523/JNEUROSCI.1674-06.2006 (2006). 16870737 PMC6674217

[b45] Feng G., Steinbach J. H. & Sanes J. R. Rapsyn clusters neuronal acetylcholine receptors but is inessential for formation of an interneuronal cholinergic synapse. The Journal of neuroscience: the official journal of the Society for Neuroscience 18, 4166–4176 (1998). 9592096 10.1523/JNEUROSCI.18-11-04166.1998 PMC6792822

[b46] Vincler M. A. & Eisenach J. C. Knock down of the alpha 5 nicotinic acetylcholine receptor in spinal nerve-ligated rats alleviates mechanical allodynia. Pharmacology, biochemistry, and behavior 80, 135–143, 10.1016/j.pbb.2004.10.011 (2005). 15652389

[b47] Gray R., Rajan A. S., Radcliffe K. A., Yakehiro M. & Dani J. A. Hippocampal synaptic transmission enhanced by low concentrations of nicotine. Nature 383, 713–716, 10.1038/383713a0 (1996). 8878480

[b48] Genzen J. R. & McGehee D. S. Short- and long-term enhancement of excitatory transmission in the spinal cord dorsal horn by nicotinic acetylcholine receptors. Proceedings of the National Academy of Sciences of the United States of America 100, 6807–6812, 10.1073/pnas.1131709100 (2003). 12748382 PMC164528

[b49] Miletic G. & Miletic V. Increases in the concentration of brain derived neurotrophic factor in the lumbar spinal dorsal horn are associated with pain behavior following chronic constriction injury in rats. Neuroscience letters 319, 137–140 (2002). 11834312 10.1016/s0304-3940(01)02576-9

[b50] Phillips H. S. . BDNF mRNA is decreased in the hippocampus of individuals with Alzheimer’s disease. Neuron 7, 695–702 (1991). 1742020 10.1016/0896-6273(91)90273-3

[b51] Lamb P. W., Melton M. A. & Yakel J. L. Inhibition of neuronal nicotinic acetylcholine receptor channels expressed in Xenopus oocytes by beta-amyloid1-42 peptide. Journal of molecular neuroscience: MN 27, 13–21, 10.1385/JMN:27:1:013 (2005). 16055943

[b52] Suberbielle E. . DNA repair factor BRCA1 depletion occurs in Alzheimer brains and impairs cognitive function in mice. Nature communications 6, 8897, 10.1038/ncomms9897 (2015). PMC4674776 26615780

[b53] Knafo S. . PTEN recruitment controls synaptic and cognitive function in Alzheimer’s models. Nature neuroscience, 10.1038/nn.4225 (2016). 26780512

[b54] Ridgway R. L., Syed N. I., Lukowiak K. & Bulloch A. G. Nerve growth factor (NGF) induces sprouting of specific neurons of the snail, Lymnaea stagnalis. Journal of neurobiology 22, 377–390, 10.1002/neu.480220406 (1991). 1890421

[b55] Syed N. I., Bulloch A. G. & Lukowiak K. *In vitro* reconstruction of the respiratory central pattern generator of the mollusk Lymnaea. Science 250, 282–285 (1990). 2218532 10.1126/science.2218532

[b56] Pfaffl M. W., Horgan G. W. & Dempfle L. Relative expression software tool (REST) for group-wise comparison and statistical analysis of relative expression results in real-time PCR. Nucleic acids research 30, e36 (2002). 11972351 10.1093/nar/30.9.e36 PMC113859

[b57] Schot L. P. & Boer H. H. Immunocytochemical demonstration of peptidergic cells in the pond snail Lymnaea stagnalis with an antiserum to the molluscan cardioactive tetrapeptide FMRF-amide. Cell and tissue research 225, 347–354 (1982). 7105153 10.1007/BF00214687

[b58] Gray E. G. & Whittaker V. P. The isolation of nerve endings from brain: an electron-microscopic study of cell fragments derived by homogenization and centrifugation. Journal of anatomy 96, 79–88 (1962). 13901297 PMC1244174

[b59] Abrams H. D., Rohrschneider L. R. & Eisenman R. N. Nuclear location of the putative transforming protein of avian myelocytomatosis virus. Cell 29, 427–439 (1982). 6288259 10.1016/0092-8674(82)90159-3

