# Supplementary material for: Two proteolytic fragments of menin coordinate the nuclear transcription and postsynaptic clustering of neurotransmitter receptors during synaptogenesis between Lymnaea neurons
Source: Sci Rep. 2016 Aug 19;6:31779. doi: 10.1038/srep31779 (PMC4990912; doi:10.1038/srep31779)
Supplement: Supplementary Information [file srep31779-s1.pdf]

SUPPLEMENTARY INFORMATION FOR

Two proteolytic fragments of menin coordinate the nuclear transcription and postsynaptic clustering of neurotransmitter receptors during synaptogenesis between *Lymnaea* neurons

AUTHORS

Angela M. Getz, Frank Visser, Erin M. Bell, Fenglian Xu, Nichole M. Flynn, Wali Zaidi, & Naweed I. Syed

This File Contains:

Supplementary Data: Tables S1 to S6 and Figures S1 to S5

Supplementary Experimental Procedures: Tables S7 to S9

SUPPLEMENTARY DATA

Supplementary Table S1. Related to Figure 2. ICC fluorescence of menin in LPeD1.

Treatment	Mean Fluorescence ± SEM (AU)	*P* (Relative to DM)	Relative Fluorescence ± SEM (Relative to DM)	n
DM	418.98 ± 15.84	(F=14.465; *P*=0.002)	1.00 ± 0.05	4
CM	674.88 ± 36.93	0.001	1.61 ± 0.07	4
DM + *MEN1* mRNA	606.28 ± 44.99	0.011	1.45 ± 0.08	4

Supplementary Table S2. Related to Figure 3. ICC fluorescence of Myc-tagged menin in LPeD1.

Treatment	Relative Fluorescence ± SEM (Nuclear Myc/Cytoplasmic Myc)	*P* (Relative to CM)	n
CM	1.25 ± 0.11	(F=24.610; *P*<0.001)	10
CM + 20 µM PD150606	0.67 ± 0.09	0.003	7
DM	0.49 ± 0.03	<0.001	10

Supplementary Table S3. Related to Figure 4. Subcellular distribution of menin in VD4-LPeD1 axon-axon pairs.

Treatment	Incidence (n)	χ^2 (Relative to CM)	*P* (Relative to CM)
Nuclear			
CM + 0.1% DMSO	5/28	-	-
DM	0/12	2.449	0.118
CM + 20 µM PD150606	3/18	0.011	0.917
CM + 40 µM U0126	0/12	2.449	0.118
CM + 1 µM KN-93	1/12	0.598	0.440
CM + 1 µM KN-92	2/17	0.299	0.585
CM + 1 µM Ch Cl^-	0/15	3.031	0.082
DM + 100 nM PMA	1/17	1.313	0.252
Non-Localized			
CM + 0.1% DMSO	4/28	-	-
DM	10/12	17.603	<0.001
CM + 20 µM PD150606	14/18	18.544	<0.001
CM + 40 µM U0126	10/12	17.603	<0.001
CM + 1 µM KN-93	8/12	10.975	0.001
CM + 1 µM KN-92	4/17	0.618	0.432
CM + 1 µM Ch Cl^-	7/15	5.380	0.020
DM + 100 nM PMA	12/17	14.634	<0.001
Synaptic			
CM + 0.1% DMSO	19/28	-	-
DM	2/12	8.827	0.003
CM + 20 µM PD150606	1/18	17.305	<0.001
CM + 40 µM U0126	2/12	8.827	0.003
CM + 1 µM KN-93	3/12	6.234	0.013
CM + 1 µM KN-92	11/17	0.047	0.828
CM + 1 µM Ch Cl^-	8/15	0.882	0.348
DM + 100 nM PMA	4/17	8.318	0.004

Supplementary Table S4. Related to Figure 5. Incidence of VD4-LPeD1 excitatory synapse formation and EPSP amplitudes.

Treatment	Incidence of Excitatory Synapses (n)	χ^2 (Relative to CM)	*P* (Relative to CM)
DM + H_2O	0/14	29.524	<0.001
CM + H_2O	35/43	-	- [1]
DM + N-*MEN1* mRNA	7/17	9.384	0.002 [1]
DM + C-*MEN1* mRNA	10/13	0.126	0.722 [1]
DM + N-*MEN1* + C-*MEN1* mRNA	10/20	6.593	0.010 [1]
DM + *MEN1* mRNA	10/15	1.387	0.239 [1]
CM + N-*MEN1* mRNA	10/13	0.126	0.722 [1]
CM + C-*MEN1* mRNA	9/12	0.240	0.624 [1]
CM + N-*MEN1* + C-*MEN1* mRNA	7/8	0.173	0.677 [1]
CM + *MEN1* mRNA	8/9	0.292	0.589 [1]
Treatment	Mean EPSP Amplitude ± SEM (mV)	*P* (Relative to CM)	n
DM + H_2O	ND [2]	-	0
CM + H_2O	10.57 ± 0.67	(F=21.64; *P*<0.001)	35
DM + N-*MEN1* mRNA	4.38 ± 0.83	0.001	7
DM + C-*MEN1* mRNA	5.50 ± 0.65	<0.001	10
DM + N-*MEN1* + C-*MEN1* mRNA	7.61 ± 1.03	0.338	10
DM + *MEN1* mRNA	7.20 ± 0.86	0.101	10
CM + N-*MEN1* mRNA	10.71 ± 0.43	1.000	10
CM + C-*MEN1* mRNA	18.51 ± 1.90	0.044	9
CM + N-*MEN1* + C-*MEN1* mRNA	20.71 ± 2.09	0.031	7
CM + *MEN1* mRNA	18.56 ± 1.76	0.033	8

1. Differences were also significant relative to DM + H_2O (Chi-squared test), *P*<0.05-0.001
2. ND indicates excitatory synapses were not detected

Supplementary Table S5. Related to Figure 6. EPSP amplitudes of VD4-LPeD1 synapses and incidence of excitatory nAChR expression in single LPeD1.

Treatment (VD4-LPeD1)	Incidence of Excitatory Synapses (n)	χ^2 (Relative to CM)	*P* (Relative to CM)
CM + 0.1 % DMSO	11/12	-	-
CM + 20 µM PD150606	11/20	4.693	0.030
DM + *MEN1* mRNA + 20 µM PD150606	2/14	15.476	<0.001
Treatment (VD4-LPeD1)	Mean EPSP Amplitude ± SEM (mV)	*P* (Relative to CM)	n
CM + 0.1 % DMSO	11.28 ± 0.92	(F=33.42; *P*<0.001)	11
CM + 20 µM PD150606	2.78 ± 0.65	<0.001	11
DM + *MEN1* mRNA + 20 µM PD150606	1.91 ± 0.38	<0.001	2
Treatment (LPeD1)	Incidence of Excitatory Receptors (n)	χ^2 (Relative to DM)	*P* (Relative to DM)
DM + H_2O	1/13	-	-
CM + 0.1 % DMSO	11/12	17.629	<0.001
CM + 20 µM PD150606	10/13	12.764	<0.001 [1]
DM + *MEN1* mRNA	6/10	7.304	0.007 [1]
DM + *MEN1* mRNA + 20 µM PD150606	9/14	9.258	0.002 [1]

1. Differences were also not significant relative to CM + 0.1% DMSO (Chi-squared test), *P*>0.05

Supplementary Table S6. Related to Figure 7. Relative gene expression in LPeD1 neurons.

LPeD1 Treatment	qPCR Target	Fold-Change Expression [1]	Standard Error	*P* [2]
DM + H_2O n=3	*MEN1*	1	0.77 - 1.30	-
	nAChR C	1	0.77 - 1.30	-
	nAChR D	1	0.52 - 1.95	-
	nAChR E	1	0.89 - 1.13	-
	nAChR G	1	0.90 - 1.12	-
	nAChR J	1	0.74 - 1.38	-
	nAChR B	1	0.85 - 1.19	-
	nAChR I	1	0.58 - 1.80	-
	nAChR K	1	0.75 - 1.35	-
DM + *MEN1* mRNA n=2	*MEN1*	13,468.13	10,383.84 - 15,880.83	0.036
	nAChR C	77.11	60.19 – 99.76	0.023
	nAChR D	0.20	0.11 - 0.34	<0.001
	nAChR E	0.41	0.37 - 0.44	0.030
	nAChR G	0.91	0.82 - 1.04	0.367
	nAChR J	30,432.67	23,465.23 - 38,361.50	<0.001
	nAChR B	0.54	0.40 - 0.73	<0.001
	nAChR I	0.64	0.40 - 1.03	0.207
	nAChR K	2.90	2.28 - 3.59	0.011
DM + N-*MEN1* mRNA n=2	*MEN1*	3,264.68	2,600.10 - 4,026.46	<0.001
	nAChR C	1,458.23	1,199.52 - 1,815.40	<0.001
	nAChR D	0.21	0.11 - 0.38	0.036
	nAChR E	0.15	0.12 - 0.18	0.066
	nAChR G	0.54	0.46 - 0.63	0.093
	nAChR J	21.96	16.21 - 26.79	<0.001
	nAChR B	0.95	0.46 - 2.39	0.884
	nAChR I	0.26	0.15 - 0.47	0.066
	nAChR K	0.26	0.20 - 0.35	0.060
DM + C-*MEN1* mRNA n=2	*MEN1*	1.66	1.34 - 2.16	<0.001
	nAChR C	184.22	144.94 - 216.81	<0.001
	nAChR D	0.10	0.06 - 0.18	<0.001
	nAChR E	0.40	0.35 - 0.43	0.063
	nAChR G	4.32	4.04 - 4.78	0.033
	nAChR J	1,114.10	863.99 - 1,378.61	<0.001
	nAChR B	0.02	0.02 - 0.02	0.029
	nAChR I	ND [3]	-	-
	nAChR K	14.65	11.16 - 17.46	<0.001
CM + 0.1% DMSO n=3	*MEN1*	3,672.08	3,026.69 - 4,661.74	<0.001
	nAChR C	2,034.64	1,674.87 - 2,468.81	<0.001
	nAChR D	2.67	1.38 - 4.77	<0.001
	nAChR E	1.24	1.06 - 1.45	0.134
	nAChR G	21.67	18.19 - 26.46	<0.001
	nAChR J	163.17	128.41 - 204.93	<0.001
	nAChR B	0.72	0.30 - 2.21	0.687
	nAChR I	1.01	0.69 - 1.63	0.924
	nAChR K	40.37	26.96 - 68.60	<0.001

Supplementary Table S6 (Continued). Related to Figure 7. Relative gene expression in LPeD1 neurons.

LPeD1 Treatment	qPCR Target	Fold-Change Expression [1]	Standard Error	*P* [2]
CM +<br>20 μM PD150606<br><br>n=3	*MEN1*	749.72	581.40 - 907.07	<0.001
	nAChR C	3.07	2.48-3.67	0.075
	nAChR D	ND [3]	-	-
	nAChR E	0.82	0.72 - 0.95	0.066
	nAChR G	2.27	1.73 - 2.89	0.072
	nAChR J	19.27	14.69 - 24.30	0.060
	nAChR B	0.26	0.21 - 0.32	0.018
	nAChR I	3.55	2.32 - 5.61	0.060
	nAChR K	8.15	6.45 - 10.46	<0.001
DM +<br>*MEN1* mRNA<br>20 μM PD150606<br><br>n=2	*MEN1*	19,222.26	14,796.54 - 24,588.00	<0.001
	nAChR C	0.007	0.00 - 0.02	0.020
	nAChR D	0.185	0.10 - 0.30	0.011
	nAChR E	0.43	0.39 - 0.48	0.041
	nAChR G	1.985	1.10 - 2.94	0.095
	nAChR J	24.525	17.59 - 36.44	<0.001
	nAChR B	0.016	0.01 - 0.02	<0.001
	nAChR I	ND [3]	-	-
	nAChR K	3.148	2.35 - 4.33	<0.001

1. Fold-change expression relative to LPeD1 – DM + H_2O
2. Statistical significance (*P*) relative to LPeD1 – DM + H_2O
3. ND indicates the transcript was not detected in the qPCR reaction.

```
                                                  NES1
Mouse       -MGLKAAQKTLFPLRSIDDVVRLFAAEL-GREEPDLVLLSLVLGFVEHFLAVNRVIPTNV 58
Lymnaea     MAGFRDRAKKHFPLTDISSVINLFKEQLEGPDEPNLALLSIVLGCIENTLTLNRAISTNE 60
              *::   *. *** .*..*:.**  :* * :**:*.***:*** :*: *::**.*.**

Mouse       PELTFQPSPAPDPPGGLTYFPVADLSIIAALYARFTAQIRGAVDLSLYPREGGVSSRELV 118
Lymnaea     DDRLLRP-----------IFPVVDLSTVDALYTKFETLVKGSVDLTKYT--GTFSSRELV 107
             :  ::*             ***.*** : ***::* : ::*:***: *.  * .******

Mouse       KKVSDVIWNSLSRSYFKDRAHIQSLFSFIT-----GTKLDSSGVAFAVVGACQALGLRDV 173
Lymnaea     KKISDVIWSSLSRS-FKDKAHLQSLYSFLT-----GNKLDCFGVAFGVVAASQLLGRNDI 161
            **:*****.***** ***:**:***:**:*     *.***. ****.**.*.* ** .*:

Mouse       HLALSEDHAWVVFGPNGEQTAEVTWHGKGNEDRRGQTVNAGVAERSWLYLKGSYMRCDRK 233
Lymnaea     HLSLSEDHAWVVFGEDGTDTAEVTWHGKGNEDKRGQSIALSVAEKSWLYLNGHPVICDRN 221
            **:*********** :* :*************:***::  .***:*****:*  : ***:

                                    NES2 Leucine Zipper
Mouse       MEVAFMVCAINPSIDLHTDSLELLQLQQKLLWLLYDLGHLERYPMALGNLADLEELEPTP 293
Lymnaea     MEVAALVSAANPSINMMTDSIEMGALQQELLWLLYDKGHLLKYPMGLGNLGDLEEISPNP 281
            **** :*.* ****:: ***:*:  ***:******* *** :***.****.****:.*.*

                                                  L-NLS
Mouse       GRPDPLTLYHKGIASAKTYYQDEHIYPYMYLAGYHCRNRNVREALQAWADTATVIQDYNY 353
Lymnaea     GRPPPLAIFQEAISSAIKYYDNHHVYPYTYMGGYLYRKRRYKEAIKCWAEASNVIRKFNY 341
            *** **:::::.*:** .** :.*:*** *:.**  *:*. :**::.**:::.**:.:**

Mouse       CREDEEIYKEFFEVANDVIPNLLKEAASLLETGEERTGEQAQGTQGQGSALQDPECFAHL 413
Lymnaea     TREDEEIYKEFLEISNELIPNIVKAVS--LNTTDR----------VQMNILYNPEVYGDI 389
             **********:*::*::***::* .:  *:: :.           * . * :** :..:

                Conserved ROI
Mouse       LRFYDGICKWEEGSPTPVLHVGWATFLVQSLGRFEGQVRQKVHIVSREAEAAEAEEP--- 470
Lymnaea     LRFYDGICEWEEGSSTPVLHVGWAQHLTFSLNKFDPRTRAKIDVGKDEDEDGEKEENDEN 449
            ********:*****.********* .*. **.:*: :.* *: : . * * .* **

Mouse       --------------------------------------------------WGDEAREGRR 480
Lymnaea     DNESEARLKEELKTDNRNKLLQSKEGKDPLKTVLEDLKKEDSSYLTQSNLAGQRVTAKNR 509
                                                              *:..   .*

            NLS1
Mouse       RGPRR-------------------------------------------------ESKPEEPP 493
Lymnaea     KGQRRRNSNASKDISTKVSKDSSRKGSIDESSKVEDKLKSKIEELVNKVGQQEGSQDATP 569
            :* **                                                 *.. : .*

Mouse       PPKKPALDKG-----------PGSGQSAGSGPP---------------RKTSGTVPG--- 524
Lymnaea     NPNITALAQQCSLSILNKDYLLGAGEPFSTSPATISTSVPADETYTTTSNTTGNFTGPDS 629
             *: .** :              *:*:    :.*.                :..*...*

Mouse       ------------------------------------TTRGGQEVGNAAQAPAPAASPPPE 548
Lymnaea     RLDVDEFLSSKSNGTAFIGLTMDSMLKAESPSDLMLAIKRSDDAARTAPLASPSPSPTAE 689
                                                : :    : . :*  .:*:.**..*

                           NLSa
Mouse       -----GPV-LTFQSEKMKGMKELLVATKINSSAIKLQLTAQSQVQMKKQKVSTPSDYTLS 602
Lymnaea     MLAALGPVYVELKSEKMKGLKKMFKSAKLNASAIKLQLTAQSQVHVKDSRFLDFCEPTGS 749
                 *** : ::******:*::: ::*:*:*************::*..:.   .: * *

              NLS2
Mouse       FLKRQRKGL- 611
Lymnaea     ARKRPRREIV 759
              ** *: :
```

Supplementary Figure S1. Related to Figure 2. The menin conserved ROI

Alignment of *Lymnaea* (accession no. AF395538) and mouse menin (accession no. NM001168488), generated with Clustal Omega (EMBL-EBI). The C-terminal epitope recognized by the α-menin antibody used in this study is underlined in green. The conserved ROI is shaded green. This stretch of 24 highly conserved residues occurs upstream of the *Lymnaea* menin sequence expansion that accounts for the size difference observed for *Lymnaea* and mammalian menin fragments (WB lower bands, see Fig. 2). Locations of known functional sequences of menin (NLS, light blue bars; NES, dark blue bars; leucine zipper motif, shaded red) are also shown.

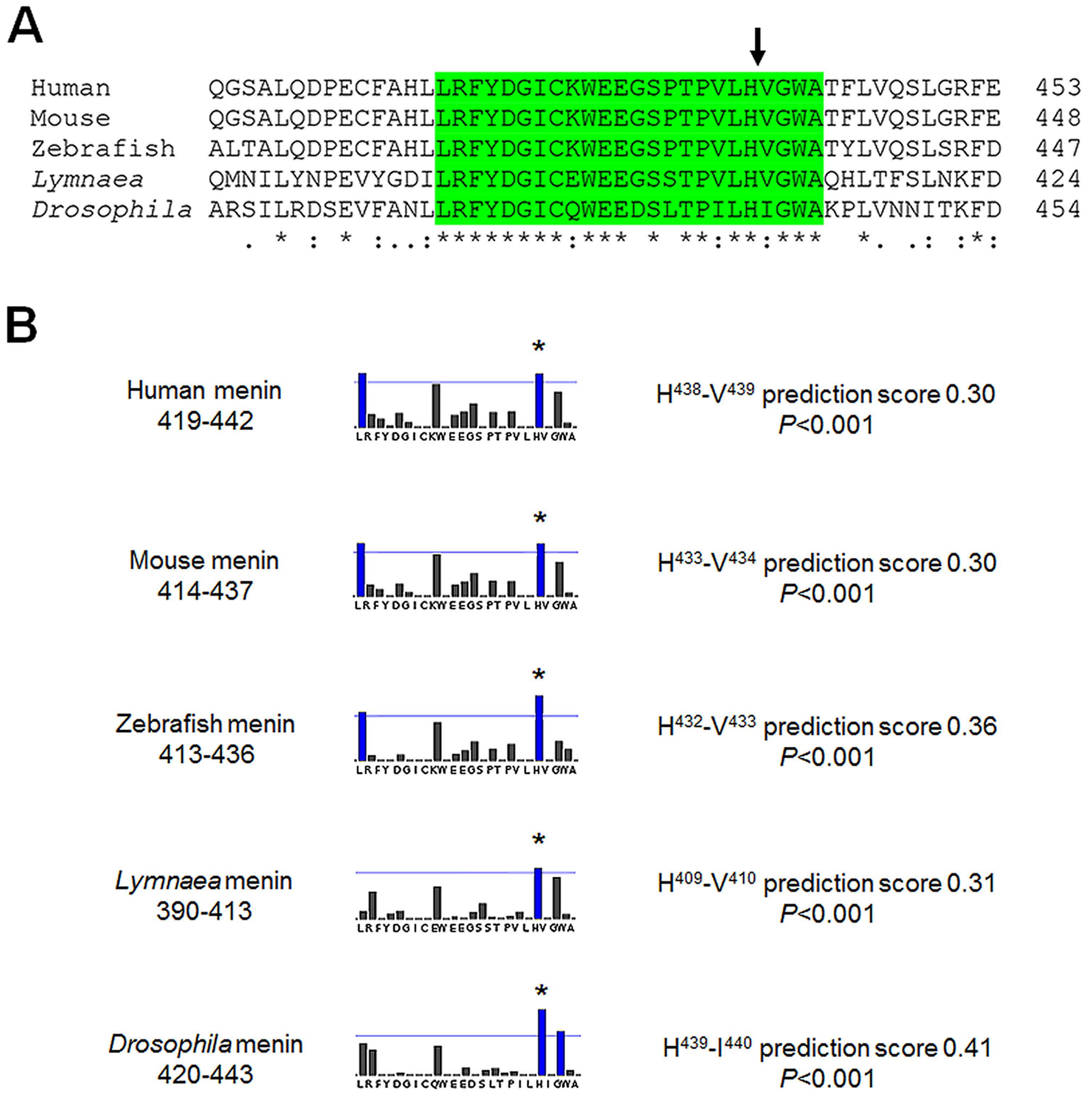

Supplementary Figure S2. Related to Figure 2. Predicted calpain cleavage site in the menin ROI is conserved

(**A**). Sequence alignment of the menin conserved ROI (shaded green) from vertebrate species human, mouse, and zebrafish, and invertebrate species *Lymnaea* and *Drosophila*. (**B**). Multiple Kernel Learning prediction identifies a presumptive calpain cleavage site in the conserved ROI of all sequences (arrow in A). Blue line indicates threshold for significant prediction scores, blue bars indicates predicted calpain cleavage sites. Asterisks indicate statistical significance of the conserved site, *P*<0.001.

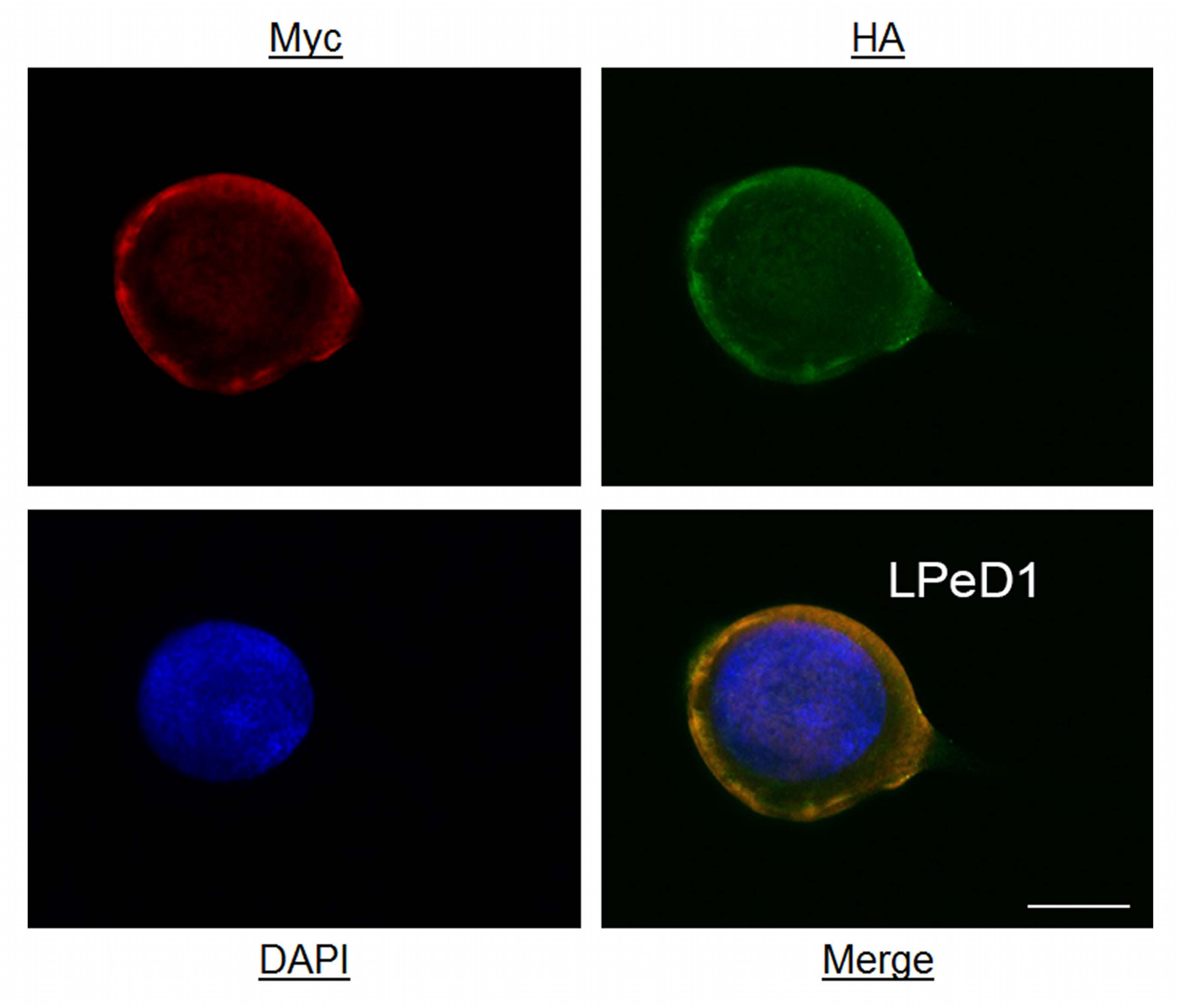

Supplementary Figure S3. Related to Figure 3. Negative control for Myc-*MEN1*-HA mRNA microinjection and epitope ICC

Single LPeD1 neurons were cultured in DM and microinjected with H_2O (vehicle control). ICC labeling of Myc and HA was performed (n=15). In the absence of Myc and HA epitopes a background signal indicative of non-specific cell surface labeling was observed (although see Fig. S4). The non-specific signals were mostly overlapping, membrane-limited, and distinct from the distribution observed with mRNA positive samples. Scale bar, 20 μm.

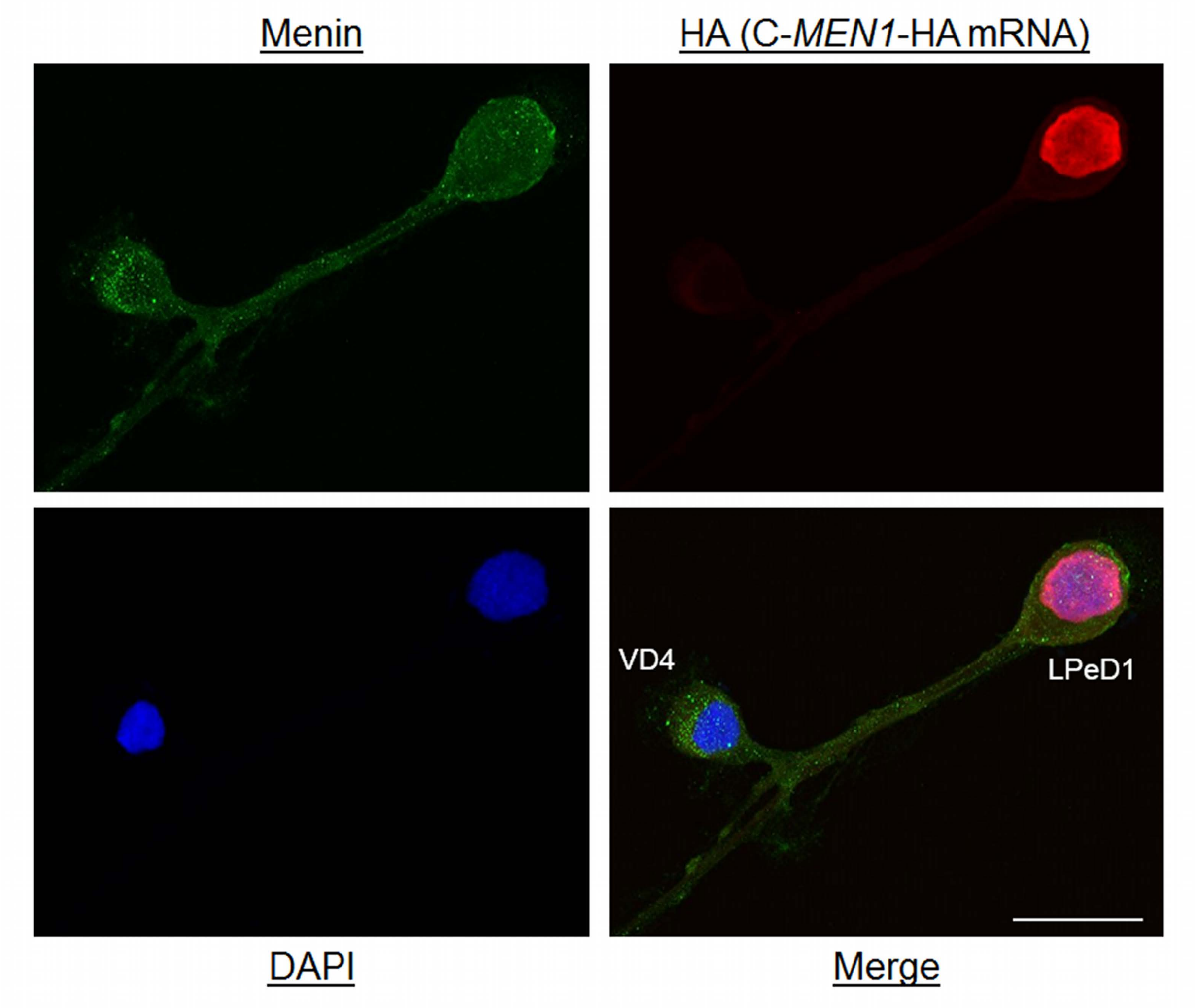

Supplementary Figure S4. Related to Figure 5. Synthetic mRNA-induced menin expression is specific to microinjected neurons

Axon-axon paired VD4-LPeD1 neurons were cultured in CM, and LPeD1 was microinjected with HA-tagged C-*MEN1* mRNA (n=3). ICC was used to detect endogenous menin (α-menin) and the C-menin fragment (α-HA). HA epitope tagged C-menin is detected only in the LPeD1 neuron. Note that nuclear localization of the C-menin fragment is observed coincident with a ‘non-localized' distribution pattern of endogenous menin, where synaptic recruitment has not been promoted by CM. Scale bar, 50 μm.

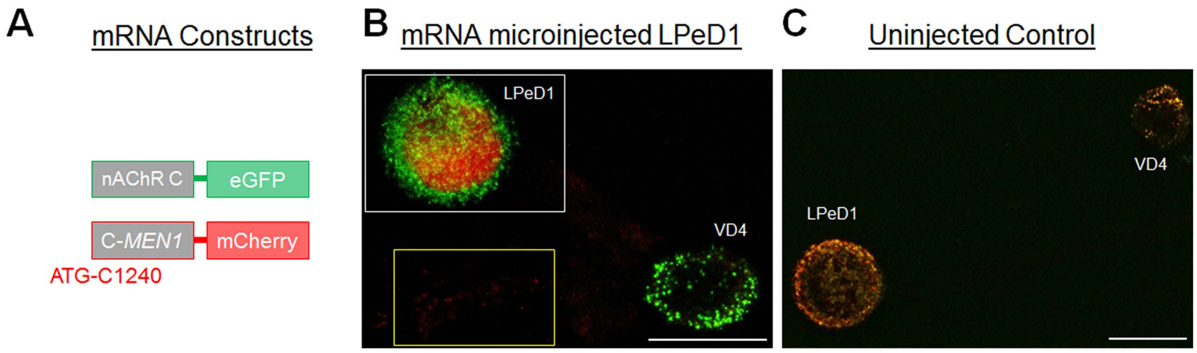

Supplementary Figure S5. Related to Figure 7. C-MEN1-mCherry and nAChRC-eGFP mRNA microinjection and live-cell imaging

(**A**). Schematic of C-*MEN1*-mCherry and nAChRC-eGFP constructs. (**B**). Merged field of view image of the axon-axon paired VD4-LPeD1 neurons depicted in Fig. 7. White boxed area is the nuclear ROI depicted in Fig. 7D. Yellow boxed area is the axonal ROI depicted in Fig. 7E. (**C**). Merged field of view image of un-injected axon-axon paired VD4-LPeD1 neurons maintained in DM for 24h (n=3). Auto-fluorescence was observed in neuronal soma but not axons. Scale bars, 50 µm.

SUPPLEMENTARY EXPERIMENTAL PROCEDURES

Supplementary Table S7. Cloning primers.

Construct	Target	Primer Sequence
Myc-*MEN1*-HA	*L*-Menin with N-terminal c-Myc and C-terminal HA epitope tags	5'GATGATCTCGAGATGGAGCAGAAGCTGATCTCAGAGGAGGACCTG GCGGGCTTTCGAGACCGAG
		3'GATGATGCGGCCGCCTAAGCGTAATCTGGAACATCGTATGGGTAGACTATTTCTCTCCTTGGCC
N-*MEN1*	*L*-Menin C-terminal truncation (residues 1-413)	5'GATGATCTCGAGATGGAGCAGAAGCTGATCTCAGAGGAGGACCTG GCGGGCTTTCGAGACCGAG
		3'GATGATGCGGCCGCCTATGCCCATCCAACATGGAGC
C-*MEN1*	*L*-Menin N-terminal truncation (residues 414-759)	5'GATGATCTCGAGATGCAGCACCTAACATTCTCTCTC
		3'GATGATGCGGCCGCCTAAGCGTAATCTGGAACATCGTATGGGTAGACTATTTCTCTCCTTGGCC
C-*MEN1*-mCherry	C-menin (residues 414-759) with C-terminal mCherry tag	5'GATGATCTCGAGATGCAGCACCTAACATTCTCTCTC
		3'CCATGTTATCCTCCTCGCCCTTGCTCACCATCCCAGACCCAGACCCAGACCCGACTATTTCTCTCCTTGGCCGTTTTCGTGC
mCherry	C-menin (residues 414-759) with C-terminal mCherry tag	5'GCACGAAAACGGCCAAGGAGAGAAATAGTCGGGTCTGGGTCTGGGTCTGGGATGGTGAGCAAGGGCGAGGAGGATAACATGG
		3'GATGATGGATCCTTACTTGTACAGCTCGTCCATGC
nAChR C-eGFP	*L*-nAChR C with C-terminal eGFP tag	5'GATGATCTCGAGATGGATGTGCTGACCAGCGC
		3'GATGATGAATTCCCCAGACCCAGACCCGTATATACATGTGTTATTTGGATC
eGFP	*L*-nAChR C with C-terminal eGFP tag	5'GATGAATTCATGGTGAGCAAGGGCGAGG
		3'GATGGATCCCTAGCTACTAGCTAGTCGAG

Supplementary Table S8. RT-PCR gene specific primers.

Target	Accession Number	5' Primer Sequence	3' Primer Sequence
β Tubulin	X15542	TCCTACTTTGTGGAATGGATCC	ATGACGAGAATTATGTCATTAGAC
18s rRNA	Z73984	CTGGTTGATCCTGCCAGTAG	CTTCCGCAGGTTCACCTAC
L-MEN1	AF395538	TCGAGACCGAGCGAAGAAAC	TTTCGTGCAGATCCTGTTGG
L-nAChR B	DQ167345	GGCCTTGACCTGCACTTACC	CATTCGCGGGCTAGGTACTC
L-nAChR C	DQ167346	CCAGCGCCATTTTCTTCTTC	TGCCACAGAAGCAAGCTGTT
L-nAChR D	DQ167347	CCTCACGGACAATGGCAGTA	GGTGTTTCCGGTTTCGTCAT
L-nAChR E	DQ167348	TAGTGCCAAGCGGTTGTACG	TATCTGGGCGGATGTTGAGA
L-nAChR G	DQ167350	GGCTCACCATGGAACAACAA	GCAACAAACTGCCACTCTGC
L-nAChR I	DQ167352	GTGTGCTTCCTGCTTGTGGT	CCCTAATGTTCGTGGCCTTC
L-nAChR J	DQ167354	AGGTTGGGATGGCAGAAGTG	CCCGATGAGAGTGACCAACA
L-nAChR K	DQ167353	GGATTCATTGATGGCTTCAACA	TGACGCACAGTGAGGTGATG

Supplementary Table S9. qPCR gene specific primers.

Target	Accession Number	5’ Primer Sequence	3’ Primer Sequence	Efficiency (%)
β Tubulin	X15542	ATCCAGGAGCTCTTCAAGCG	CTGTGAACTCCATCTCGTCC	105.80
18s rRNA	Z73984	CACGGGGAGGTAGTGACG	GCCCTCCAATGGGTCCTC	103.40
L-MEN1	AF395538	TGGAGTTCGCTGTCTCGAAG	CAAAGGCAACACCAAAGCAA	93.52
L-nAChR B	DQ167345	GCCAATGTCTGCAGCAGAC	GTCGCTTTGTTCCTGCACGG	100.42
L-nAChR C	DQ167346	GAGACGGACATGATCAAGCC	CATAGGTCCTGCCGACGGC	109.04
L-nAChR D	DQ167347	GGCCTCACAGGACTACCAAC	GGTCAGAGGCGTTGTACACG	106.21
L-nAChR E	DQ167348	GAGGAGGAGTGGCTACAAC	CATGATTTGGTTCTTCTCGTC	102.55
L-nAChR G	DQ167350	GACCAAGTCTTGGTTTCTGG	GATAGGAGGAGCCAATGAGG	108.01
L-nAChR I	DQ167352	GTACCGCTTCCAGTGATATC	GTTGACCACTGGCCTGATG	93.52
L-nAChR J	DQ167354	GGAAGGACTACCAGCTGGAG	CATCGGCATTGTTGAAAAGCAC	106.21
L-nAChR K	DQ167353	CTTCCGGCGTAGGTCCACC	GCAGGTGATTCTGGAGGTATC	96.06
